# Functional reprogramming of monocytes in patients with acute and convalescent severe COVID-19

**DOI:** 10.1172/jci.insight.154183

**Published:** 2022-04-05

**Authors:** Elisa Brauns, Abdulkader Azouz, David Grimaldi, Hanxi Xiao, Séverine Thomas, Muriel Nguyen, Véronique Olislagers, Ines Vu Duc, Carmen Orte Cano, Véronique Del Marmol, Pieter Pannus, Frédérick Libert, Sven Saussez, Nicolas Dauby, Jishnu Das, Arnaud Marchant, Stanislas Goriely

**Affiliations:** 1 Institute for Medical Immunology (IMI) and ULB Center for Research in Immunology (U-CRI), ULB, Brussels, Belgium.; 2 Intensive Care Unit, Hôpital Erasme, Brussels, Belgium.; 3 Center for Systems Immunology, Department of Immunology, Department of Computational and Systems Biology, University of Pittsburgh School of Medicine, Pittsburgh, Pennsylvania, USA.; 4 Department of Dermatology, Hôpital Erasme, Brussels, Belgium.; 5 SD Epidemiology and Public Health, Sciensano, Brussels, Belgium.; 6 Institute of Interdisciplinary Research (IRIBHM) and Brightcore, ULB, Brussels, Belgium.; 7 Department of Otolaryngology, Epicura, Mons, Belgium.; 8 Department of Infectious Diseases, Hôpital Saint-Pierre, Brussels, Belgium.

**Keywords:** COVID-19, Immunology, Cytokines, Imprinting, Monocytes

## Abstract

Severe COVID-19 disease is associated with dysregulation of the myeloid compartment during acute infection. Survivors frequently experience long-lasting sequelae, but little is known about the eventual persistence of this immune alteration. Herein, we evaluated TLR-induced cytokine responses in a cohort of mild to critical patients during acute or convalescent phases (*n* = 97). In the acute phase, we observed impaired cytokine production by monocytes in the patients with the most severe COVID-19. This capacity was globally restored in convalescent patients. However, we observed increased responsiveness to TLR1/2 ligation in patients who recovered from severe disease, indicating that these cells display distinct functional properties at the different stages of the disease. In patients with acute severe COVID-19, we identified a specific transcriptomic and epigenomic state in monocytes that can account for their functional refractoriness. The molecular profile of monocytes from recovering patients was distinct and characterized by increased chromatin accessibility at activating protein 1 (AP1) and MAF loci. These results demonstrate that severe COVID-19 infection has a profound impact on the differentiation status and function of circulating monocytes, during both the acute and the convalescent phases, in a completely distinct manner. This could have important implications for our understanding of short- and long-term COVID-19–related morbidity.

## Graphical abstract

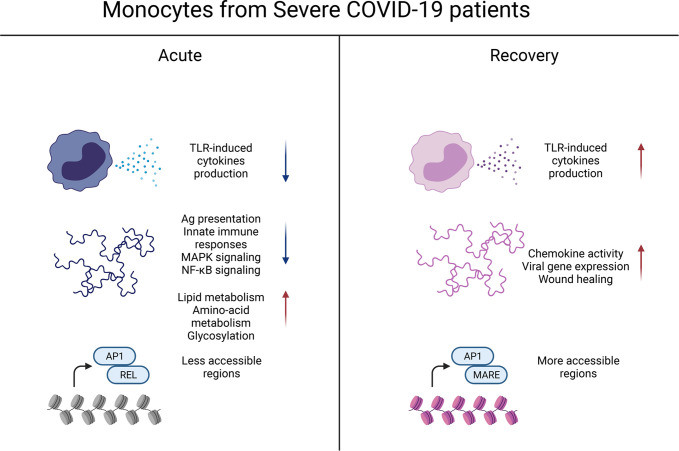

## Introduction

COVID-19, caused by the SARS-CoV-2 virus primarily infects the respiratory tract. There is a very broad spectrum of disease presentation. Some people who get COVID-19 have only mild symptoms. But for others, infection leads to pneumonia, respiratory failure, and, in some cases, death. The major complication of severe COVID-19 infection is acute respiratory distress syndrome (ARDS) presenting with dyspnoea and acute respiratory failure that requires mechanical ventilation. Severe COVID-19 appears to be associated with coagulopathy presenting as thrombosis in various organs, and it is proposed that SARS-CoV-2 causes lesions to endothelial cells — a process that then triggers inflammation throughout the body and fuels ARDS (1, 2). Multiple studies indicate that the mononuclear phagocyte system is strongly perturbed during acute infection and contributes to this hyperinflammatory complication (3–6). In patients with mild COVID-19, there is a specific increase of inflammatory monocytes that display a strong IFN-stimulated gene signature. This was shown to be absent in severe disease. Instead, in these patients, there are signs of emergency myelopoiesis, marked by the occurrence of immunosuppressive neutrophils and HLA-DR^lo^ monocytes (7, 8). Impaired type I IFN production and decreased expression of HLA-DR by plasmacytoid and conventional DCs, respectively, was also reported in these patients (9, 10).

In view of the novel nature of this disease, little is known about the long-term residual deficits of these patients. Initial assessment of COVID-19 survivors indicate that this disease could have multiple long-term effects leading to respiratory but also cardiovascular, renal, or neurological sequelae (11, 12). Here, in the light on the complex immune dysregulation that occurs during acute infection (6, 13), we hypothesized that COVID-19 could have long-term impact on immune functions, as it is observed in patients who experienced bacterial sepsis (14). Indeed, immune profiling of SARS-CoV-2–recovered patients indicates persistent changes in the phenotype of innate (monocytes and granulocytes) and, most importantly, adaptive (T, B, and NKT) immune cells (15–20).

In this study, we explore the functional features of circulating monocytes in patients with acute and convalescent with COVID-19. Stimulation with TLR ligands associated with bacterial or viral patterns established the functional impairment of circulating mononuclear phagocytes in acutely ill and severely affected patients. This was not observed in convalescent patients, but we observed a distinct pattern of cytokine production. We further evaluated the transcriptional and epigenomic profiles of CD14^+^ monocytes from patients who experienced severe COVID-19. Taken together, these data support an underlying epigenetic basis for functional reprograming of monocytes during and after severe COVID-19.

## Results

### Characteristics of the enrolled individuals.

To assess the eventual persistence of immune dysregulation, we enrolled patients during acute infection and during the convalescence phase (2–9 months after the onset of the symptoms, secondarily divided into “early recovery” for days 56–120 and “late recovery” for days 160–268 after symptoms onset). Patients were categorized according to the severity of the disease: “mild disease” represents patients who were not hospitalized, and “severe disease” corresponds to patients who required hospitalization due to COVID-19 (in regular ward [severe group] or intensive care unit [ICU] critical group). Hospitalized patients were more frequently male and older (Table 1). Most patients (97%) had a nasopharyngeal reverse transcription PCR (RT-PCR) positive for SARS-CoV-2 at the hospital admission; otherwise, the diagnosis of COVID-19 was based on clinical, serological, and radiological features. During the acute phase, none of the patients with mild COVID-19 died, while death occurred in 38% of hospitalized patients. Comorbidities and other demographic and disease features are shown in extended data (Supplemental Table 1; supplemental material available online with this article; https://doi.org/10.1172/jci.insight.154183DS1).

### Increased levels of inflammatory cytokines in hospitalized patients.

**Figure 1:**
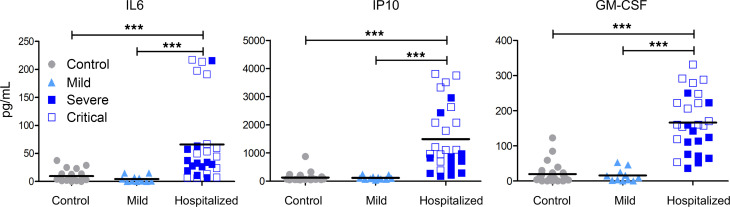
Increased levels of inflammatory cytokines in hospitalized patients during acute infection. Measurements of cytokine and chemokine levels in the plasma of controls (*n* = 32), as well as mild (*n* = 11) and hospitalized patients (severe,*n* = 7; critical, *n* = 17) during acute infection. Kruskal-Wallis test was performed to examine the statistical differences between groups, followed by Dunn’s correction for multiple testing. ***P < 0.001. Each dot represents an individual donor, and bars represent the mean values.

As described in several studies (21–23), we observed increased circulating levels of inflammatory cytokines such as IL-6, IP10, and GM-CSF in hospitalized patients compared with patients with mild COVID-19 (Figure 1) — in particular, if they had needed admission to ICU. This validates the clinical division of our patients.

### Strong alteration of TLR-induced cytokine production by classical monocytes in hospitalized patients during acute infection.

To evaluate the functionality of the innate immune cells in COVID-19, we performed ex vivo stimulation of whole blood with different pathogen-associated molecular patterns (PAMPs): bacterial LPS, a TLR4 ligand; R848, a synthetic ligand for TLR7/8; and Pam3CSK4 (PAM), a synthetic ligand for TLR1/2. Medium alone served as unstimulated condition. We performed multiplex determination of cytokine levels in the supernatants and assessed cytokine production in monocytes at the single-cell level by multiparametric flow cytometry.

**Figure 2:**
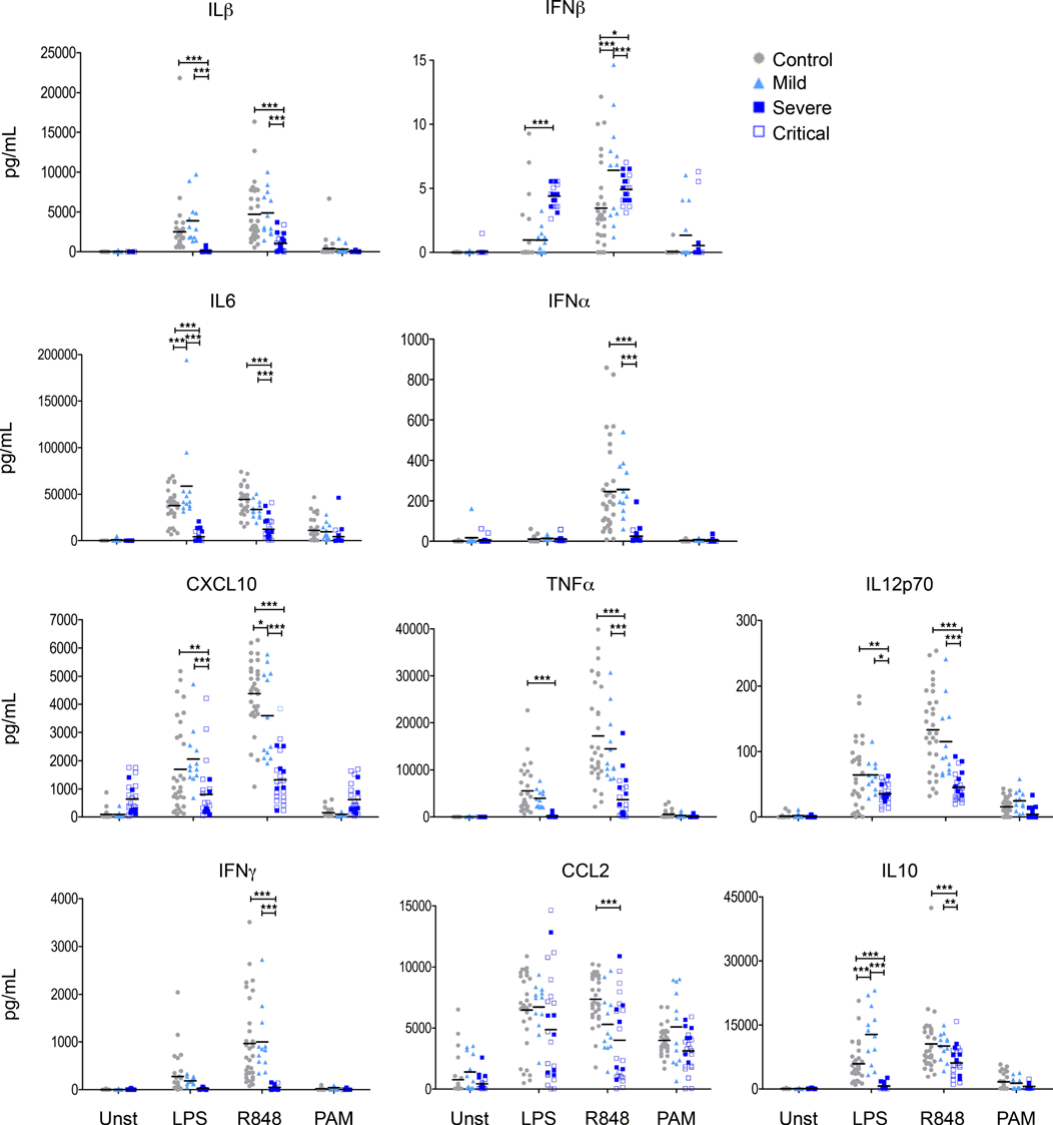
Reduced TLR-induced cytokines levels during acute severe COVID-19. Measurements of cytokine and chemokine levels in the supernatant of whole blood culture after stimulation for 24 hours in controls (*n* = 32), as well as mild (*n* = 11) and hospitalized patients (severe, *n* = 7; critical, *n* = 17) during acute infection. Two-way ANOVA was performed to examine the statistical differences of each cytokine per group and per stimulation, followed by Bonferroni post hoc tests. *P < 0.05, ***P < 0.001. Each dot represents an individual donor, and bars represent the mean values.

**Figure 3:**
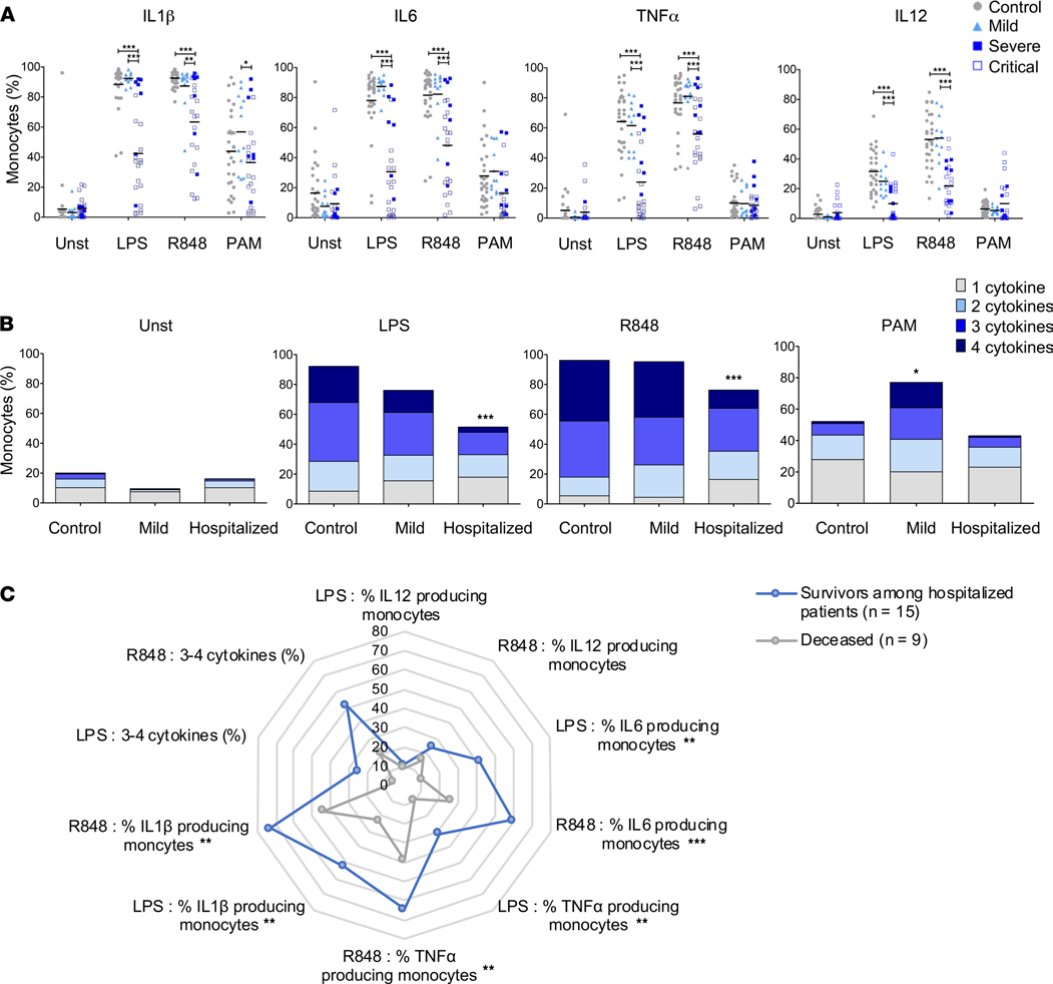
TLR-induced cytokine production by CD14^+^ monocytes is impaired during acute severe COVID-19. (**A**) Flow cytometry analysis of intracellular production of cytokines by CD14^+^ monocytes upon 6-hour stimulation of whole blood from controls (*n* = 32), as well as mild (*n* = 11) and hospitalized patients (severe, *n* = 7; critical, *n* = 17) during acute infection. Two-way ANOVA was performed to examine the statistical differences of each cytokine/monocyte per group and per stimulation, followed by Bonferroni post hoc tests. Each dot represents an individual donor, and bars represent the mean values. (**B**) Measure of the ability of monocytes to produce up to 4 cytokines among TNF-α, IL-6, IL-12p40, and IL-1β simultaneously (polyfunctionality) upon stimulation among acute patients. Kruskal-Wallis test was performed to examine the statistical differences in the ability to produce 3 or 4 cytokines simultaneously per group, followed by Dunn’s correction for multiple testing. Statistical significance in comparison with controls is indicated. (**C**) Radar chart of cytokines production and polyfunctionality upon LPS and R848 stimulation, according to the outcome among hospitalized patients. *P < 0.05, **P < 0.01, ***P < 0.001.

In acutely ill patients who did not require hospitalization, we observed few significant modulations of cytokine production in comparison with healthy controls: increased IFN-β levels in response to R848 and increased LPS-elicited IL-10 and IL-6 production. In sharp contrast, in hospitalized patients, we observed strongly reduced levels of almost all cytokines in response to LPS or R848 stimulation, with the exception of IFN-β, which was significantly increased (Figure 2). These alterations tended to be worse in patients requiring ICU admission. Of note, in comparison with other stimuli, PAM induced low levels of some cytokines (CCL2, IL-6, IL-10), and we did not observe significant differences between the groups. Because whole blood is a complex mixture of cells that can directly or indirectly produce these cytokines in response to TLR stimulation, we also evaluated cytokine production at the single-cell level using flow cytometry. Consistent with our Luminex data, the proportion of CD14^hi^ monocytes expressing IL-1β, IL-6, TNF-α, and IL-12/23 in response to LPS or R848 was found to be decreased in hospitalized patients in comparison with healthy subjects or patients with mild disease (Figure 3A). Critical patients were more severely affected than patients hospitalized in the regular ward. In contrast to these conditions, stimulation with PAM was weaker but was comparable in the 3 groups.

To fully apprehend the extent of this functional alteration, we were interested in evaluating the capacity of monocytes to produce more than 1 cytokine simultaneously (i.e., polyfunctionality). The capacity to produce 3 or 4 cytokines concurrently was impaired in hospitalized patients during acute COVID-19 in response to stimulation by LPS or R848 but not by PAM (Figure 3B). This latter parameter was increased in monocytes from patients with mild COVID-19 as compared with controls. Taken together, these experiments demonstrate that the ability of monocytes to mount an appropriate proinflammatory response is impaired in patients with severe COVID-19 upon stimulation through TLR4 or TLR7/8. When considering the most informative immune parameters in all patients with acute COVID-19, we observed a very strong correlation between the different responses to LPS and R848, as compared with the same analysis in naive subjects. This indicates that, during SARS-CoV-2 infection, modulation of the capacity of monocytes to produce different cytokines is highly coordinated and that the effect is global (Supplemental Figure 1A).

Based on these data for our 24 patients with severe COVID-19, we defined whether these immune parameters were correlated with clinical outcome. As shown in Figure 3C, a low degree of cytokine production was significantly associated with a higher risk of death among hospitalized patients, indicating that innate immune paralysis is a key feature of the most severe forms of COVID-19.

### Functional modulation of monocyte function in severe COVID-19 convalescent patients.

**Figure 4:**
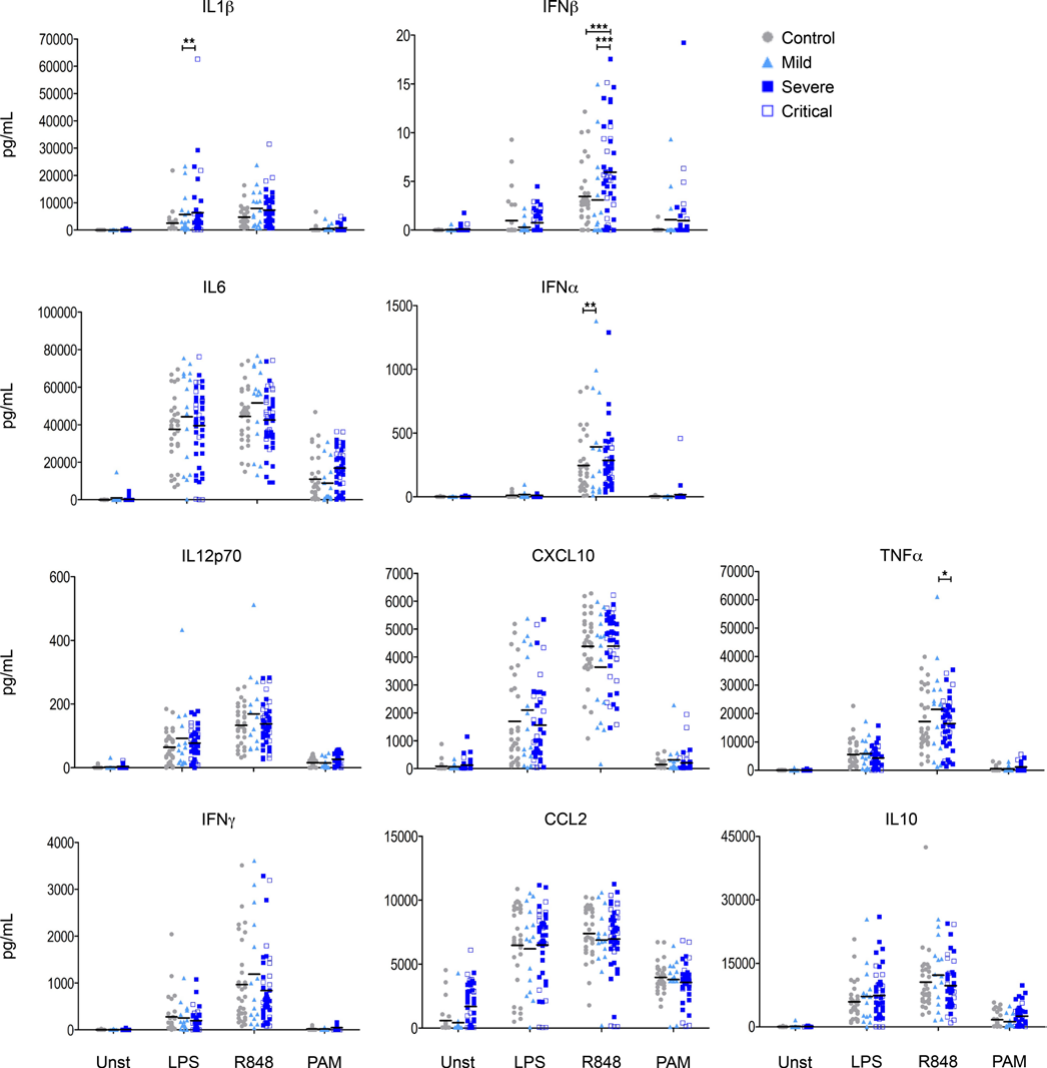
Normalization of TLR-elicited cytokine levels in whole blood from convalescent patients. Measurements of cytokine and chemokine levels in the supernatant of whole blood culture after stimulation for 24 hours in controls (*n* = 32), as well as mild (*n* = 16) and hospitalized (severe, *n* = 28; critical, *n* = 18) patients during recovery phase. Two-way ANOVA was performed to examine the statistical differences of each cytokine/monocyte per group and per stimulation, followed by Bonferroni post hoc tests. *P < 0.05, **P < 0.01, ***P < 0.001. Each dot represents an individual donor, and bars represent the mean values.

In order to define whether this immune dysfunction persists after the resolution of the acute infectious episode, we prospectively recruited patients 56–268 days after the onset of the symptoms. We quantified cytokine production in whole blood assays. Except for increased IFN-β concentrations in previously infected and hospitalized subjects, cytokine levels were found to be comparable in noninfected controls and convalescent patients, indicating that the global dysfunctional state observed during severe COVID-19 infection is not long-lasting (Figure 4). Of note, although it did not reach statistical significance in the whole group, we also observed high basal CCL2 production in a subset of these patients. In patients recovering from mild COVID-19, there was a trend for higher IL-1β, IL-6, and TNF-α production.

**Figure 5:**
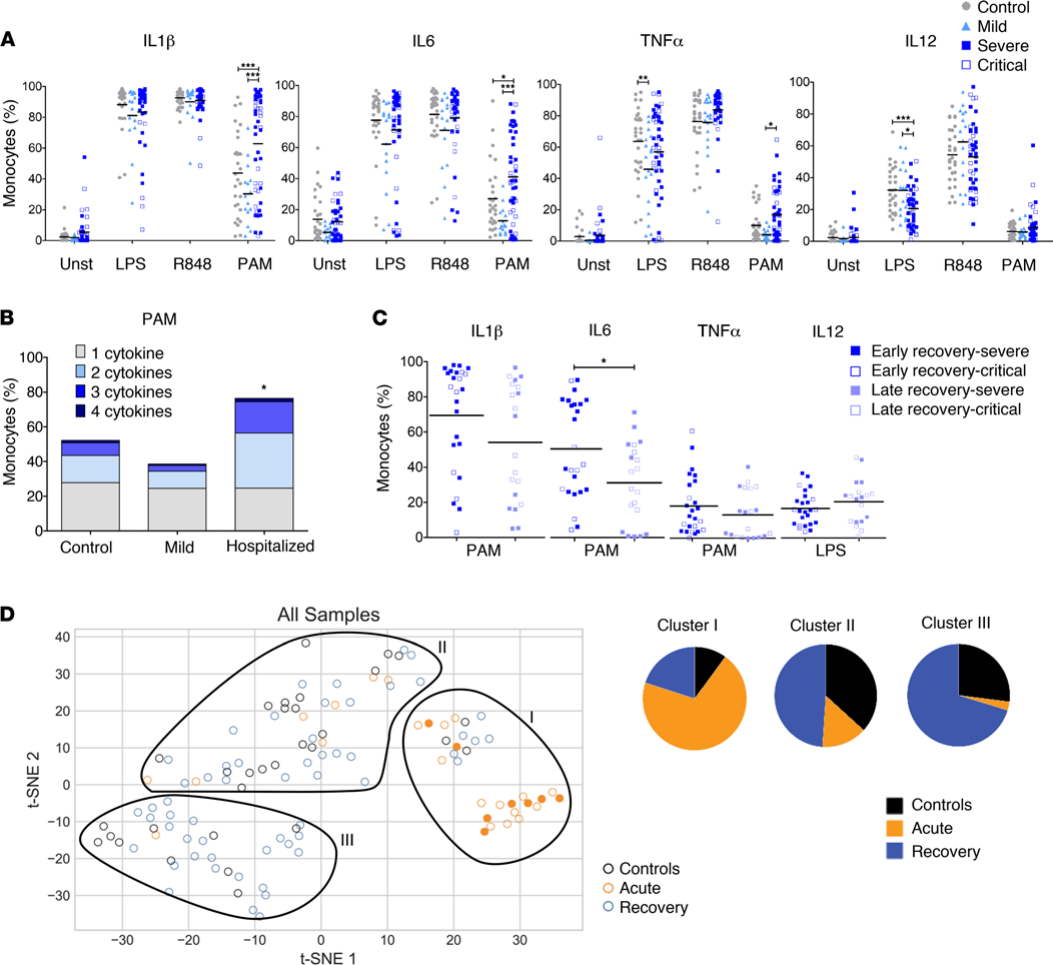
Modulation of cytokine expression in classical monocytes during recovery phase. (**A**) Flow cytometry analysis of intracellular production of cytokines by CD14^+^ monocytes upon 6-hour stimulation of whole blood from controls (*n* = 32), as well as mild (*n* = 16) and hospitalized (severe, *n* = 28; critical, *n* = 18) patients during recovery phase. Two-way ANOVA was performed to examine the statistical differences of each cytokine/monocyte per group and per stimulation, followed by Bonferroni post hoc tests. Each dot represents an individual donor, and bars represent the mean values. (**B**) Measure of the ability of monocytes to produce up to 4 cytokines among TNF-α, IL-6, IL-12p40, and IL-1β simultaneously (polyfunctionality) upon PAM stimulation during early recovery phase. Kruskal-Wallis test was performed to examine the statistical differences in the ability to produce 3 or 4 cytokines simultaneously per group, followed by Dunn’s correction for multiple testing; statistical difference is expressed compared with controls. (**C**) Flow cytometry analysis of intracellular production of cytokines by CD14^+^ monocytes upon 6-hour stimulation of whole blood from hospitalized patients at early recovery (*n* = 25; severe, *n* = 17; critical, *n* = 8) or late recovery stages (*n* = 21; severe, *n* = 11; critical, *n* = 10). A Mann-Whitney *U* test was performed to examine the statistical differences. Each dot represents an individual donor, and bars represent the mean values. (**D**) t-SNE plot of all stimulated samples according to the phase of the disease; filled dots represent deceased patients. Proportions in each manually gated cluster is represented in the right panel. *P < 0.05, ***P < 0.001.

Of note, focusing on classical monocytes, patients who recovered from a severe disease showed an increased capability to produce IL-1β, IL-6, and TNF-α upon engagement of TLR1/2. On the other hand, in response to LPS stimulation, we noted a reduced proportion of IL-12/23^+^ cells in the same group of patients (Figure 5, A and B). This distinctive pattern appeared to concern mainly patients in the early phase of recovery (Figure 5C).

To visualize how all the different immune parameters vary across the clinical groups, we used t-distributed stochastic neighbor embedding (t-SNE), an unsupervised (i.e., without using group/outcome labels) visualization approach for high-dimensional data. The t-SNE analyses help capture multivariate differences in immune parameters across the samples (Figure 5D). We used an initial feature selection to identify immune parameters that are significantly different across naive controls, patients with acute COVID-19 infection, and convalescent patients. We then performed a t-SNE on the downselected features (Supplemental Figure 1). Our analysis shows an approximate 3-way separation with clusters enriched either for patients with COVID-19 in the acute (cluster I) or convalescent stages (cluster III). Cluster I comprised a very clear subgroup of patients who were severely ill. In conclusion, alteration of TLR responsiveness is a key feature of the most severe forms of COVID-19. Months after the recovery, the monocytes of these patients displayed a distinct pattern of cytokine production, which suggests that prior COVID-19 infection may induce functional reprograming that lingers several months after recovery.

### Monocytes from patients with acute and convalescent COVID-19 display distinctive transcriptomic profiles.

**Figure 6:**
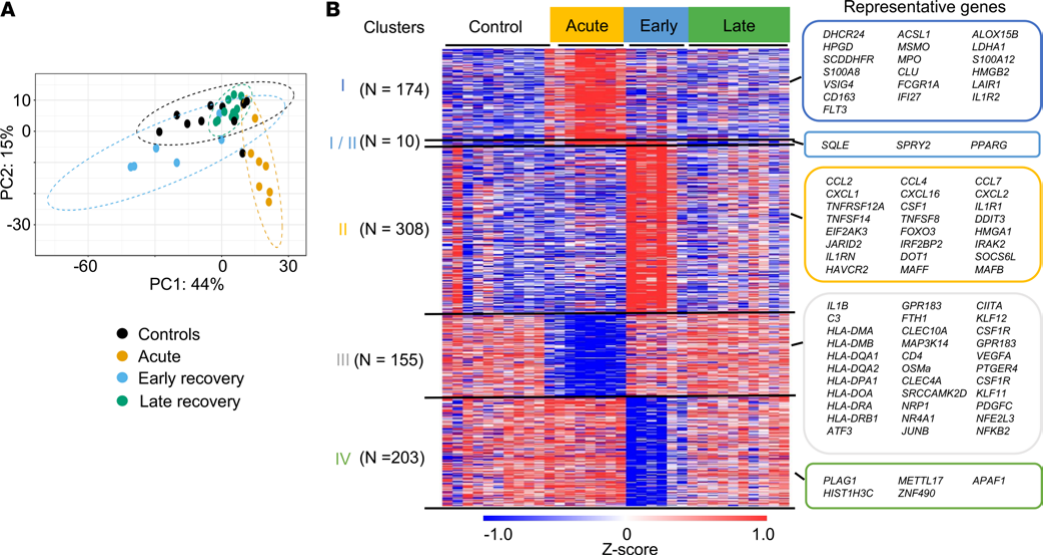
Distinct transcriptomic profiles of monocytes during acute and recovery phases. (**A**) PCA plot representing the distinct clusters based on transcriptional profiles of monocytes from controls (*n* = 11), as well as acute-infection (*n* = 7), early-recovery (days 56–120 after symptoms onset, *n* = 6), and late-recovery phases (days 160–268 after symptoms onset, *n* = 10). (**B**) Heatmap of differential genes for controls (*n* = 11), as well as acute-infection (*n* = 7), early-recovery (*n* = 6), and late-recovery phases (*n* = 10); representative genes were selected.

**Figure 7:**
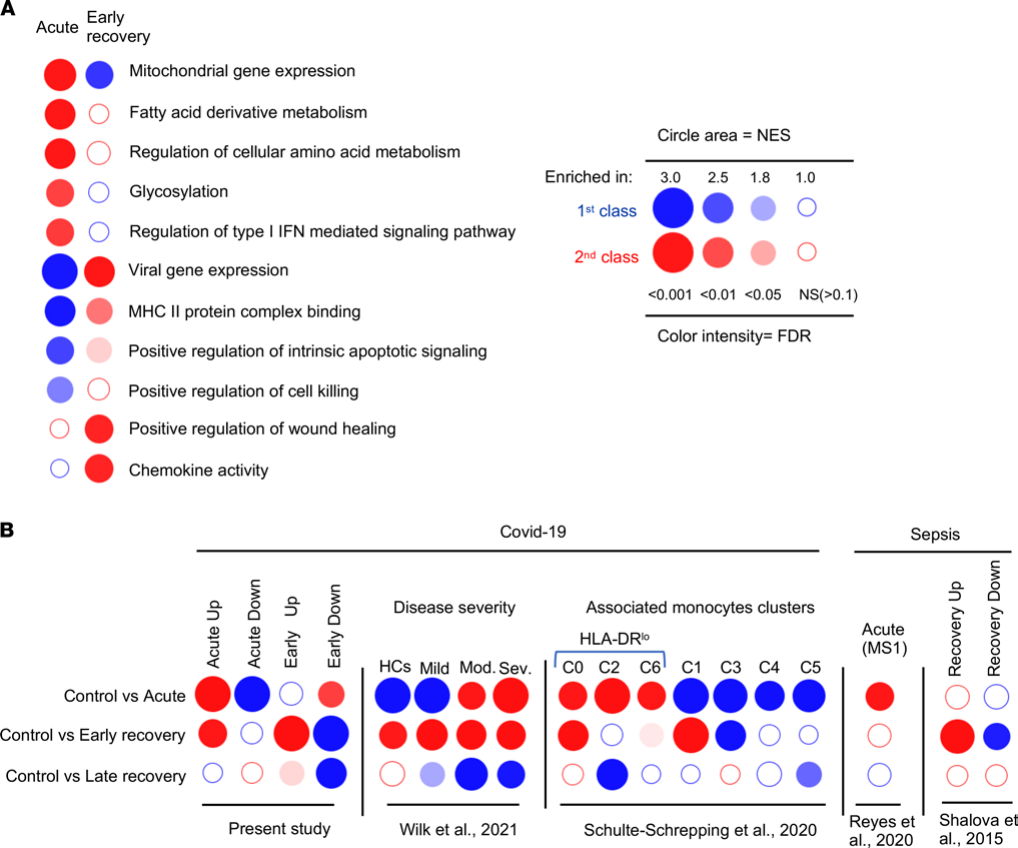
Transcriptomic profiles of monocytes from acute and recovery COVID-19 patients are characterized by distinct features. (**A** and **B**) BubbleGUM gene set enrichment analysis (GSEA) map established from GO pathways (database MsigDB; https://www.gsea-msigdb.org/gsea/msigdb/) (**A**) and from available gene sets in monocytes during COVID-19 or sepsis (**B**). For each gene set, origin of the data set and number of upregulated (red) and downregulated (blue) genes are indicated. The panel summarizes the normalized enrichment score (NES) and FDR parameters. HCs, healthy controls; Mod., moderate; Sev., severe.

To gain further insight into the molecular features of CD14^+^ monocytes in these severely affected patients with COVID-19 during acute and convalescent stages, we selected only hospitalized patients from our cohort and age- and sex-matched controls, and we performed global transcriptional profiling. Characteristics of these individuals are shown in Supplemental Table 2. Since we observed differences in terms of cytokine production between early (56–120 days after symptom onset) and late recovery stages (160–268 days after symptoms onset) (Figure 5B), we analyzed these groups separately. We observed a clear separation between samples from acute and healthy controls upon principal component analysis (PCA; Figure 6A). For samples from patients who recovered from COVID-19, those from early recovery stage formed a distinct cluster, while those from a late recovery stage were found to be embedded within the control group. In acute samples, we identified 339 statistically differentially expressed genes (DEG: 184 up- and 155 downregulated genes compared with controls, with a fold change [FC] > 2 and a FDR < 0.05; Figure 6B). Consistent with their altered functional response and recent reports (24, 25), we observed decreased expression of genes related to key immune pathways, such as antigenic presentation, innate immune responses and MAPK and NF-κB signalling (*JUNB*, *ATF3*, *NFkB2*) in monocytes from patients suffering from severe COVID-19 (Figure 6B). We also observed increased expression of genes involved in key metabolic processes, including lipid metabolism (Figure 7A). Finally, we evaluated the expression of genes associated with monocytic myeloid-derived suppressor cells (M-MDSC) (26, 27). M-MDSC–like cells represent an immature HLA-DR^lo^CD14^+^ population with immunosuppressive properties, and this has been described in various pathological situations, including severe COVID-19 (28). Although we observed decreased expression of MHC II–related genes, this notion was not supported by our data, as the expression levels of immunosuppressive genes such as *ARG1*, *IL-10*, or *IDO1* was not increased during acute severe disease (Supplemental Figure 2).

Using the same criteria for samples from convalescent patients in the early recovery period, we identified 521 DEGs (318 up- and 203 downregulated genes compared with controls). Expression of multiple genes encoding chemokines was upregulated, along with important intracellular immunomodulatory proteins and transcription factors (*PPARG*, *FOSL1*, *MAFB*, *MAFF*, *ATF4*, *FOXO3*). Hardly any DEGs were identified for the patients at the latter stage. Importantly, very few genes that were up- or downregulated in the acute stage were found to be also modulated in the recovery groups (Figure 6B), indicating that the transcriptomic program induced by the acute infection is not persistent. We performed flow cytometry staining for selected surface markers that had been identified as differentially expressed genes in RNA-Seq experiments (Figure 6B) in hospitalized patients: CD163 and IL-1R2 for cluster I, TIM3 (CD366) for cluster II, and HLA-DR for cluster III. As previously described, HLA-DR expression by CD14^+^ monocytes was significantly downregulated during acute severe infection; conversely, CD163 was upregulated in a subset of patients in the same group. IL-1R2 and CD366 were increased in a subset of acute and early recovery patients, respectively, but without reaching statistical significance (Supplemental Figure 3).

We observed enrichment for distinct pathways during the acute and the recovery phase (Figure 7A). For instance, during the acute phase, multiple pathways involved in cell metabolism were strongly enriched, while wound healing and chemokine activities were identified in the recovery phase.

We also analyzed publicly available gene sets from single-cell RNA-Seq (scRNA-Seq) data of patients with acute COVID-19 (Figure 7B). In the acute phase, we observed strong enrichment for genes identified in monocytes from the most severely affected patients (29). Schulte-Schrepping et al. described several blood monocyte subsets that arise in patients with mild and severe COVID-19 (7). In monocytes from acute samples, we observed strong enrichment for marker genes of cluster C2 (HLA-DR^lo^CD163^hi^ cells) that corresponds to the cells that are the most abundant in early stages of the disease in patients with severe COVID-19. In monocytes from recovered patients, the strongest enrichment was seen for marker genes of cluster C0 (HLA-DR^lo^S100A^+^ cells) that dominates later in the course of the acute disease and of cluster C1 (HLA-DR^hi^CD83^+^ cells) that corresponds to activated cells. Monocytes from acute patients were also enriched for the MS1 gene signature derived from immature monocyte state in sepsis patients (30). In contrast, genes that were modulated in the recovery phase (i.e., several weeks after the sepsis) (31) were significantly up- or downregulated in early-recovery patients, indicating potential common underlying mechanisms between these 2 clinical situations. Altogether, our transcriptomic data on CD14^+^ monocytes from patients with acute severe COVID-19 indicate that these cells display an altered profile that could account for their decreased responsiveness to PAMPs. The distinctive transcriptional state of monocytes identified in recovery patients is probably not related to the same processes that occur in acutely ill patients and is not persistent in later stages of convalescence. This unique profile displays similarities with monocytes from patients who recovered from sepsis.

### Monocytes from patients with acute and convalescent severe COVID-19 display distinct profiles of chromatin accessibility.

**Figure 8:**
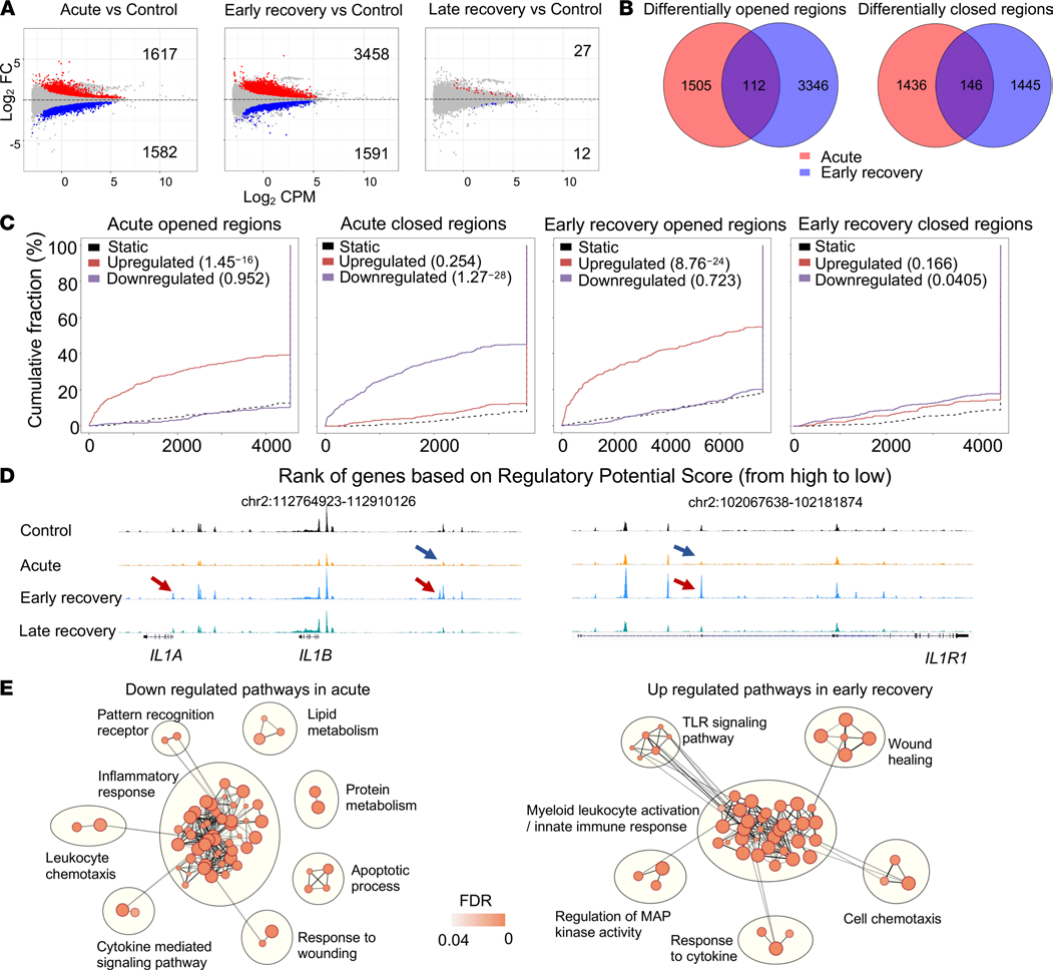
Epigenetic reprogramming of monocytes in patients with acute and convalescing severe COVID-19. (**A**) MA plot (M [log ratio] and A [mean average]) showing log_2_ fold change (FC) average read density of differentially accessible regions (DARs) in CD14^+^ monocytes of controls (blue) and patients at the different phases of the disease (red), with the indicated number of regions. (**B**) Venn diagrams showing the intersection between the indicated comparisons. (**C**) Cumulative distribution plot generated by BETA algorithm showing the predicted activating/repressive function of more or less accessible enhancer regions in monocytes during acute and early recovery phases, and the indicated *P* values determined by the Kolmogorov-Smirnov test (red, upregulated genes; blue, downregulated genes; dashed line, background). (**D**) Representative ATAC-Seq tracks of CD14^+^ monocytes for the different groups, with less (blue arrow) or more (red arrow) accessible peaks in comparison with controls. Position of each locus in the genome is indicated at the top of each track. (**E**) Gene set enrichment network displays clusters of redundant pathways associated with closing regions in acute (left) or opening regions in early recovery patients (right). Nodes represent gene sets, and edges represent mutual overlap. Overlap significance is indicated by the edge’s thickness. Color denseness indicates the normalized enrichment score (NES).

To identify the epigenetic determinants of these distinct transcriptional and functional programs, we mapped chromatin accessibility by Assay for transposase accessible chromatin sequencing (ATAC-Seq). As expected, we observed extensive modifications in monocytes from patients with acute COVID-19 as 1617 and 1582 regions were found to be more or less accessible in acute patients versus controls, respectively (Figure 8A). Strikingly, we also observed important changes during the early but not in the latter stages of recovery. A low number of differentially accessible regions (DARs) identified were common to both comparisons (Figure 8B). We used the Binding and Expression Target Analysis (BETA) package (32) to predict the activating or repressive function of these DARs. For acute patients, regulatory regions that were more or less accessible were clearly associated with genes that were up- or downregulated at the transcriptional level, respectively (Figure 8C). For early convalescent patients, we also observed a strong association between the regions that are more accessible and genes that are activated in this group. In contrast, the regions that were found to be less accessible were not clearly associated with downregulated genes, suggesting that the most important regulatory features in this case are linked to gene activation rather than repression. These observations strongly suggest that epigenetic imprinting is responsible for the transcriptional signatures identified in acute and early convalescing patients. For example, in acute patients, less accessible regions were found in the loci of *IL1B* or *IL1R1* genes (Figure 8D). In early convalescent patients, we observed increased accessibility at the same regulatory regions.

**Figure 9:**
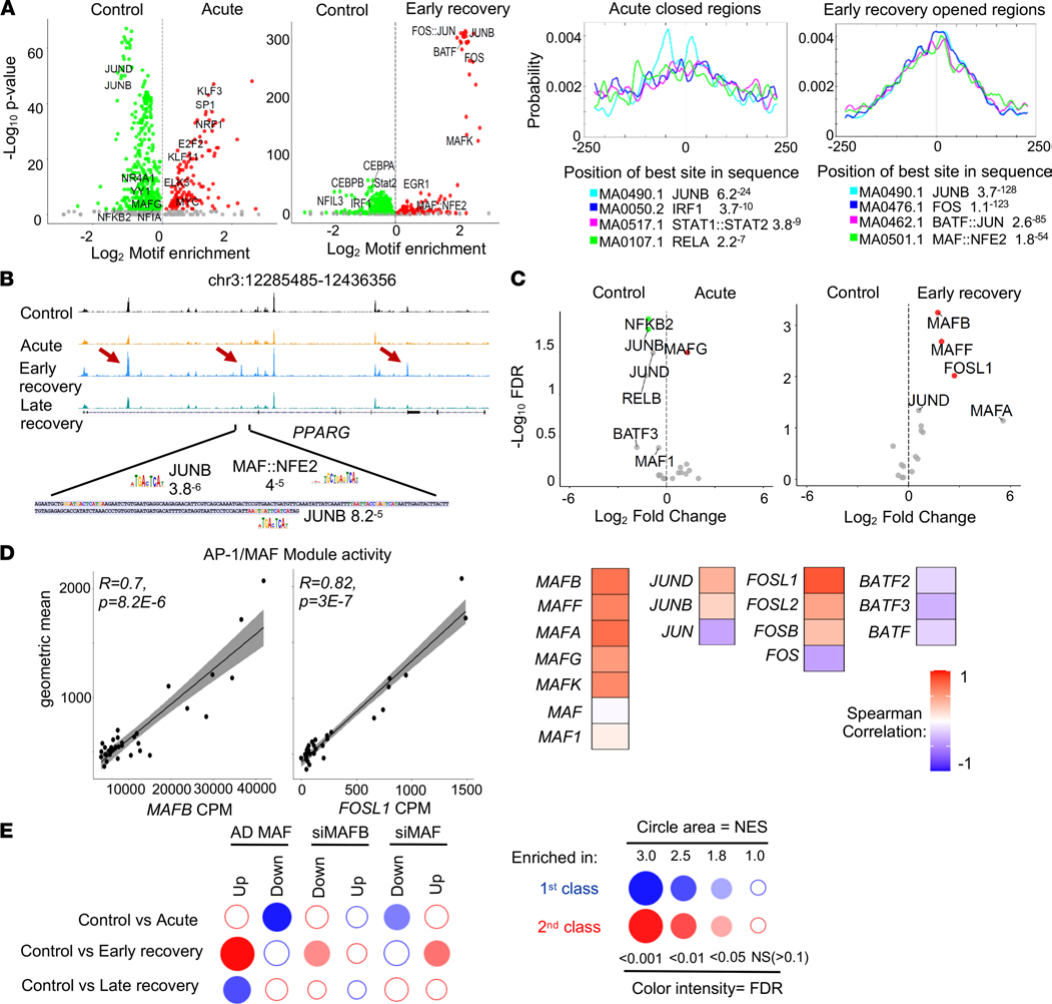
Motifs associated with differentially accessible regions in monocytes from COVID-19 patients. (**A**) CiiiDER analysis of putative transcription factor motifs enrichment (left) and representative motifs centrality (right) on differentially opened and closed regions in acute and convalescing patients. (**B**) Representative ATAC-Seq tracks at the *PPARG* locus. Peaks with increased accessibility in early recovery patients are indicated by a red arrow. Sequence of the highlighted region and the location of JUN and MAF motifs with their *P* value are indicated. (**C**) Volcano plot showing changes in expression of AP1-, NF-κB–, and MAF-related genes in acute (right) or early recovery patients (left) in comparison with controls. (**D**) Correlation between the mRNA expression of the indicated transcription factor and the AP1/MAF module activity in the whole data set. Representative plots for *MAFB* and *FOSL1* are shown. Spearman correlation coefficients for each gene are represented in a heatmap (right). (**E**) BubbleGUM gene set enrichment analysis (GSEA) of data sets from controls, as well as early-recovery and late-recovery patients. Publicly available gene sets were obtained from macrophages infected by MAF-expressing adenovirus (24) or treated with siRNA targeting either MafB or MAF (42). The panel summarizes the normalized enrichment score (NES) and FDR parameters.

Next, we performed gene ontology (GO) analysis using Genomic Regions Enrichment of Annotations Tool (GREAT) (33). Consistent with our transcriptomic data, we observed that regulatory regions that are less accessible in monocytes from acute patients were associated with genes involved in inflammatory response, lipid metabolism, or cytokine-mediated signaling pathways (Figure 8E). We observed a mirror image in recovery patients with increased accessibility associated to genes involved in innate immune response and TLR signalling pathway but also in wound healing. We then scanned for binding motifs at the center of ATAC peaks located in these sets of regions. Analysis of putative TF site enrichment in patients versus control-specific regions indicated a strong and significant enrichment for distinct motifs in both groups (Figure 9A). Consistent with the decreased expression of *NFKB2* and *JUNB* (Figure 9C), we observed an overrepresentation for activating protein 1 (AP1; Jun/Fos) and REL binding motifs in regions that were less accessible in monocytes from acute patients. We also identified IRF1 and STAT motifs in these regions, suggesting that multiple inflammatory modules are deactivated at this stage of the disease. In sharp contrast, in regions that were more accessible in convalescent patients, we observed a strong enrichment for AP1 and MAF recognition element–containing (MARE-containing) motifs (Figure 9A). We identified both motifs in a substantial proportion of these regions (845 of 3458 regions, linked to 121 significantly upregulated genes in recovery patients in comparison with controls, termed AP1/MAF transcriptional module). For example, such motifs were identified in the locus of the *PPARG* gene (Figure 9B). Of note, we observed increased levels of *MAFB* and *MAFF*, but also *FOSL1*, a partner of Jun family members that displays an important role in orchestrating the expression of genes related to wound response, TLR activation, and IL signaling of macrophages (34). Given that JUN proteins also form heterodimers with members of the MAF family (35), it is tempting to speculate that upregulation of *MAFB* expression in these cells redirects AP1 complexes to these genomic targets, thereby promoting local chromatin remodeling. Consistent with this hypothesis, we observed a strong correlation between *MAFB* and *FOSL1* levels and expression of the AP1/MAF transcriptional module in the whole cohort (Figure 9D). These correlations were less obvious with other members of the MAF, JUN, FOS, or BATF families. Finally, we observed strong enrichment for MAF-responsive genes in monocytes from convalescent patients in comparison with controls (Figure 9E) (36). MafB-dependent genes rather than cMAF-dependent genes were enriched, reinforcing the potential contribution of this specific transcription factor. Finally, we looked specifically at epigenetic regulators that were differentially expressed between convalescent patients and controls (Supplemental Figure 4A). We observed modulation of the expression of genes encoding important enzymes involved in histone modifications, such as p300 or KDM6B, that could account for changes in enhancer landscape. Along this line, expression of *EP300*, a histone acetyltransferase, and of *KDM6B*, an H3K27me3 histone demethylase, was strongly correlated with the level of accessibility measured at regulatory regions associated with the MAF/AP1 transcriptional module (Supplemental Figure 4B).

Taken together, these data support the notion that the transcriptional program and functional properties of monocytes in patients with acute or convalescing severe COVID-19 have strong epigenetic determinants.

## Discussion

Multiple reports now indicate that COVID-19 infection is associated with drastic changes in the myeloid compartment, particularly in patients with a severe course of disease (25, 37–39). It has been shown that severe and fatal COVID-19 leads to accumulation of HLA-DR^lo^ monocytes with potentially suppressive and dysfunctional features. Here, to evaluate the function of these cells more thoroughly, we first assessed the capacity of circulating cells from patients with mild and severe COVID-19 to produce cytokines in response to prototypical TLR ligands. We observed that the ability of monocytes from patients with severe COVID-19 to produce inflammatory cytokines was severely impaired. Of note, stimulation with the TLR1/2 ligand PAM was generally less affected than with other ligands, and we observed even higher levels of IFN-β in LPS- or R848-stimulated whole blood cultures, indicating complex functional changes. Moreover, among severely ill patients, decreased ability of monocytes to produce several cytokines simultaneously was associated with a higher risk of mortality.

Our transcriptomic and epigenomic analysis of CD14^+^ monocytes indicated that the functional alterations identified in patients with acute severe COVID-19 are accompanied by decreased basal activity of key modules involved in TLR signaling pathways, including NF-κB and AP1. Multiple molecular mechanisms may account for the unique phenotypic and functional features of monocytes in this context.

The intense systemic inflammation could have direct effects on circulating cells but could also trigger emergency hematopoiesis and the release of immature cells from the BM, as observed in sepsis (40). However, we did not observe a clear correlation between plasmatic levels of inflammatory cytokines and functional capacity of circulating monocytes in hospitalized patients. In addition, our data do not support the emergence of a distinct M-MDSC–like population as recently identified by flow cytometry–based criteria (28). It would nevertheless be important to further assess the functional properties of these cells and their contribution to adaptive immune responses (41). Importantly, treatment itself could also have an impact on the functional features of circulating monocytes, since a vast majority of hospitalized patients during acute phase received corticosteroids as standard of care and sometimes other immunomodulatory drugs (including hydroxychloroquine, anti-IL therapies, and antiviral drugs).

Next, to define whether this hematopoietic reprograming during acute severe COVID-19 infection could have a long-term impact on monocyte function, we recruited patients who had recovered from the disease. The production of cytokines in patients convalescing from mild or severe disease was globally comparable with that of SARS-CoV-2 naive controls. However, at the single-cell level, we observed subtle changes, including decreased production of IL-12 in response to LPS and increased responsiveness to TLR1/2 ligation in patients who recovered from severe disease. This was more obvious in the first few months after the disease, suggesting that the impact of COVID-19 infection/hospitalization on monocyte function fades after a longer period.

In these early convalescent patients (2–3 months after infection), we observed striking molecular profiles characterized by epigenomic reprogramming reminiscent of trained immunity. This term has been proposed for the persistent enhanced state of the innate immune response following exposure to certain infectious agents or vaccines, and this may result in increased resistance to related or unrelated pathogens (42). However, similar processes may also result in hyporesponsiveness and be deleterious — for example, in the context of chronic metabolic and inflammatory diseases such as liver cirrhosis, which is a condition associated with increased susceptibility to infections (43). More recently, Wimmers et al. showed that administration of a seasonal influenza vaccine induced persistent epigenomic changes in myeloid cells, leading to innate refractoriness associated with decreased AP1 activity (44). Multiple mechanisms may account for these long-lasting effects on innate immune cells. In the context of BCG immunization, exposure to LPS, or sepsis, these effects involve modulation of myeloid progenitors in the BM (45–47). In the context of COVID-19, they could also be the consequence of persistent viral antigen expression (detected up to 4 months after the onset of the disease) (48) or result from the modulation of the adaptive compartment, as the majority of SARS-CoV-2–specific CD8^+^ T cells acquire a terminally differentiated phenotype (49).

Single-cell epigenomic profiling identified distinct states among classical monocytes and indicated that “activated” or “trained” subsets were enriched in patients with convalescing COVID-19 (50). In line with this report, we observed that the epigenomic state of monocytes from patients who recovered from severe COVID-19 was associated with heightened AP1 and MAF activities. We uncovered the potential contribution of FOSL1 and MafB in this functional reprograming. FOSL1 has been shown to regulate pro- and antiinflammatory cytokine expression in macrophages, modulating profibrotic responses (51) and promoting lung or joint inflammation (34, 52). MafB is also a key regulator involved in functional programing of macrophages in the context of tissue imprinting (53), lipid/cholesterol-rich environments, or wound healing (54). Of note, the balance between MAF and MafB in alveolar macrophages could shape the response to SARS-CoV-2 (55). The epigenetic basis of these functional and transcriptional modifications is strongly suggested by the correlation between accessibility of regulatory regions associated with AP1/MAF transcriptional module and the expression of enzymes involved in histone modifications. In line with this notion, modulation of monocyte activity observed after influenza vaccination was found to be associated with global changes in H3K27ac and H3K23me3 levels (44).

This functional reprograming of monocytes in patients with convalescing COVID-19 could have both beneficial and detrimental consequences. In particular, an emerging complication of COVID-19 infection is a prolonged period of lingering symptoms after infection (12). A hyperinflammatory state seems to be a cardinal feature of this syndrome that is also associated with increased risk for sudden cardiovascular events (56). Hence, the epigenomic changes in circulating monocytes we describe here could contribute to cytokine-driven endothelial dysfunction (57).

We are mindful of the limitations of the present study. It would be important to follow these patients longitudinally, as they represent a very heterogeneous population. Hence, it is possible that more subtle variations — for example, in patients with mild COVID-19 — have been overlooked. In addition, the differences we identified here using bulk approaches probably reflect the changes in the most abundant subsets of monocytes. Furthermore, we cannot exclude a selection bias in convalescent patients who survived from an acute infection. Finally, multiple factors that could influence the function and epigenetic reprogramming of these cells were not taken into account. For example, treatment modalities evolved between the different waves, thanks to a better understanding of COVID-19 physiopathology and the results of clinical trials. The frequent occurrence of superinfections in patients requiring mechanical ventilation could impact the state of circulating immune cells (58). Comorbidities could also alter the response to the infection and its resolution. Finally, we could not include other respiratory infection as a supplemental and possibly more accurate control group.

In conclusion, it is now clear that immune responses are strongly influenced by past and present interactions of the host immune system with its environment (59). We show here that severe COVID-19 infection has a profound impact on the differentiation status and function of circulating monocytes during the acute and the convalescent phases in a completely distinct manner. This could have important implications for our understanding of short- and long-term COVID-19–related morbidity and mortality.

## Methods

### Patient recruitment.

For this study, between June 2020 and January 2021, we recruited 97 patients during acute infection or at early and late recovery phases. Patients were categorized according to the severity of the disease; “mild disease” represents patients who weren’t hospitalized, and “severe disease” corresponds to patients who were hospitalized due to COVID-19 (in regular ward or ICU). Among acute infections, 11 were mild and 24 were severe. Nineteen patients recovered from mild and 43 from severe disease. As controls, we recruited 32 SARS-CoV-2–naive individuals (negative nasopharyngeal RT-PCR and serology for SARS-CoV-2) among health care workers and nursing home residents (60) who were age- and sex-matched to patients with severe COVID-19.

### Blood collection.

All blood draws were performed in the hospital by a trained phlebotomist. Peripheral blood was drawn via sterile venipuncture into sodium-heparin vacutainers. Blood samples were kept at room temperature (RT) and processed within 4 hours of the blood draw.

For ex vivo FACS analysis, part of the samples was directly preserved using whole blood processing kit (Cytodelics) and stored at –80°C before further staining.

### PRR stimulation.

To minimize technical artifacts, we used a highly standardized, stringently controlled protocol as described previously (61). Briefly, premade 96-well plates contained the following specific PRR ligands were prepared: PAM (TLR2/1; InvivoGen) at 1 μg/mL; LPS (TLR4; InvivoGen) at 10 ng/mL; R848 (TLR7/8; InvivoGen) at 10 μM; and media alone. Whole blood, diluted 1:1 with sterile prewarmed RPMI 1640, was added to each well containing the specific TLR ligands.

For the intracellular cytokine staining (ICS), 10 μg/mL of brefeldin A (final concentration, Sigma-Aldrich) was added to each well, and samples were incubated for 6 hours at 37°C in 5% CO_2_; they were then treated with 2 mM EDTA (final concentration) for 10 minutes at 37°C. The cells were collected and resuspended in BD FACS Lysing Solution, placed into fresh tubes, and stored at –80°C.

For multiplex analysis, samples were incubated with whole blood for 24 hours at 37°C in 5% CO_2_. The supernatant was collected and stored at –80°C.

### Staining and flow cytometric acquisition.

For the ICS, frozen tubes were thawed and spun, and pellets were washed multiple times with wash buffer (PBS, 5% FCS). Cells were then stained for 30 minutes at RT with the membranal antibodies. After multiple washes, samples were incubated for 20 minutes at 4° with BD Cytofix/Cytoperm solution, before being stained for 20 minutes at RT with the intracytoplasmic antibodies. A second panel for markers selected based on transcriptomic analysis was performed. Samples were defrosted according to the manufacturer’s instructions. Cells were stained for 30 minutes at RT with the dedicated antibodies. All the antibodies used are listed in Supplemental Table 3.

Flow cytometric data acquisition was performed on a Cytoflex LX Beckmann & Coulter and analyzed using FlowJo software (see Supplemental Figure 5 for gating strategies).

### Cytokine measurement in whole blood culture supernatant and plasma.

Human XL Cytokine Luminex Performance Panel assays (Bio-Techne) were used to measure the levels of TNF-α, IL-6, IL-12p70, IL-1β, IL-10, IFN-α, IFN-β, IFN-γ, CXCL10, and CCL2 in the supernatants following stimulation and of IL-6, IP10, and GM-CSF in the plasma isolated from blood samples.

### Isolation of peripheral blood mononuclear cells (PBMCs) and sorting of CD14^+^ monocytes.

PBMCs were isolated from sodium-heparinized whole blood by density gradient centrifugation using Lymphoprep (ProteoGenix) and Leuco-Sep tubes (Greiner), and PBMCs were stored in FCS with 10% DMSO in liquid nitrogen. PBMCs were thawed, stained with trypan blue, and counted to ensure a majority of live cells. CD14^+^ monocytes were isolated by FACS on a SONY SH800S Cell Sorter (Supplemental Figure 6). Postsort purity was higher than 90%.

### RNA-Seq.

In total, 15,000–200,000 CD14^hi^ monocytes from 11 healthy controls, 13 patients with acute COVID-19, and 20 patients who recovered from COVID-19 were isolated by FACS directly in TRIzol reagent (Thermo Fisher scientific). After chloroform extraction, RNA isolation was performed using the RNeasy kit (Qiagen), and sample quality was tested on a Fragment Analyzer (Agilent). Indexed cDNA libraries were obtained using the Ovation Solo RNA-Seq System (Tecan) following manufacturer recommendations. The multiplexed libraries were loaded on a NovaSeq 6000 (Illumina) using a S2 flow cell, and 25 × 10^6^ paired-end reads/sample were produced using a 200 cycle kit. The RNA-Seq was performed by BRIGHTcore ULB-VUB. Adapters were removed with Trimmomatic-0.36 with the following parameters: Truseq3-PE.fa:2:30:10 LEADING:3 TRAILING:3 SLIDINGWINDOW:4:15 MINLEN:36 HEADCROP:4. Reads were then mapped to the reference genome GRCh38 by using STAR_2.5.3a software with default parameters. We sorted the reads from the alignment according to chromosome positions and indexed the resulting BAM files. Read counts in the alignment BAM files that overlap with the gene features were obtained using HTSeq-0.9.1 with “--nonunique all” option (if the read pair aligns to more than 1 location in the reference genome, it is counted in all features to which it was assigned and scored multiple times). Genes with no raw read count greater or equal to 20 in at least 1 sample were filtered out with an R script, raw read counts were normalized, and a differential expression analysis was performed with DESeq2 by applying an adjusted *P* < 0.05 and an absolute log_2_ ratio larger than 1.

### ATAC-Seq.

ATAC followed by sequencing was performed as follows: 10,000–50,000 sorted CD14^+^ monocytes from 11 healthy controls, 14 patients with acute COVID-19, and 21 patients who recovered from COVID-19 were collected in 1 mL of cell culture media at 4°C. Cells were centrifuged (10 minutes at 500*g* at 4°C); then, cell pellets were resuspended in 50 μL of lysis buffer (Tris HCl 10 mM, NaCl 10 mM, MgCl2 3 mM, Igepal 0.1%) and centrifuged (500 *g*) for 25 minutes at 4°C. Supernatant was discarded, and nuclei were resuspended in 50 μL of reaction buffer (Tn5 transposase 2.5 μL, TD buffer 22.5 μL, and 25 μL H_2_O; Nextera DNA sample preparation kit, Illumina). The reaction was performed for 30 minutes at 37°C. DNA was purified using the MinElute purification kit (Qiagen). Purified DNA was amplified and indexed by PCR using NEBNext High-Fidelity 2× PCR Master Mix (New England Biolabs) with 10–12 cycles. Amplified libraries were purified using MinElute PCR Purification Kit (Qiagen), followed by a double AMPURE XP purification (0.5:1 and 1.2:1 ratios) and quality controlled using a Fragment Analyzer High-Sensitivity DNA Analysis kit (Agilent). Paired-end sequencing was performed on NovaSeq platform (Illumina).

Adapters in obtained reads were removed with Trimmomatic 0.36 with the following parameters: Nextera1.fa:1:25:6 LEADING:3 TRAILING:3 SLIDINGWINDOW:4:15 MINLEN:36. Paired-end reads were mapped to human genome GRCh38 with Bowtie2 (62, 63) using the following parameters for paired-end reads: –X 2000 –fr –very-sensitive –no-discordant –no-mixed –non-deterministic. Reads from the alignment were sorted and indexed according to chromosomes. Reads located within the blacklist of the ENCODE projectDuplicate reads were removed with MarkDuplicates tools (Picard suite). Peaks were called with MACS2 (64) using the following parameters: -f BAMPE -g mm -q 0.05 --nomodel --call-summits -B –SPMR.

Regions obtained by MACS2 were merged to create an atlas containing all obtained peaks for all the populations using bedtools (65) with a minimum overlapping of 1 bp. Merged regions were subject to differential analysis using csaw workflow (66).For downstream visualization, a scaling factor was calculated using deepTools package (67) to normalize peak intensity to fraction of reads in peaks (FRiP) and generate bigwig files.

For GO analysis, we introduced BED files from differential ATAC-Seq peaks to GREAT with default parameters (33). For motif analysis, the CiiiDER algorithm (68) was used to perform motif enrichment in the differentially accessible regions.

We used BETA package (32) with default parameters to integrate ATAC-Seq (differentially accessible regions) and RNA-Seq (transcriptome) data and to evaluate the regulatory potential of chromatin accessibility to promote/repress genes expression.

### Unsupervised analysis.

To assess which immune parameters — i.e., features — best explained the differences across the clinical categories, we performed a greedy feature selection to retain immune parameters significantly different (*P* < 0.0001 using a Kruskal-Wallis test) across the categories. The centered and scaled cellular phenotypic data corresponding to patients in the different clinical categories was visualized in 2 dimensions using t-SNE with the downselected features as inputs to the t-SNE. The observed clustering was stable across t-SNE technical replicates.

### Data availability.

RNA-Seq and ATAC-Seq data that support the findings reported in this study have been deposited in the GEO Repository with the accession code no. GSE198257.

### Statistics.

Dichotomous variables were analyzed with χ^2^ test. Group comparisons were performed using 2-way ANOVA followed by Bonferroni post hoc tests or Kruskal-Wallis test with Dunn’s correction for multiple testing, when appropriate. Statistical analysis was conducted using Python, R, Prism Version 5 (GraphPad), or IBM SPSS Statistics 22. Unless otherwise specified, the level of statistical significance was set at *P* < 0.05.

### Study approval.

The protocol was approved by the local ethics committee (Epicura, Baudour, Belgium: P2020011; Erasme, Brussels, Belgium: B4062020000029; CHU Saint-Pierre, Brussels, Belgium: CE200910) and was conducted according to the guidelines of the 1975 Declaration of Helsinki. Written informed consent was obtained from all patients or their designated family members.

## Author contributions

EB conducted most of the experiments. EB, VDM, PP, and AM elaborated the clinical studies. ND, DG, COC, and SS contributed to the recruitment of the patients and the controls. AA, VO, MN, ST, IVD, and FL contributed to some experiments. AA performed bioinformatics analysis. EB and AA analyzed the data and prepared the figures. JD and HX provided input for data analysis and interpretation. EB and SG wrote the manuscript. SG designed and supervised the work. All authors were involved in critically revising the manuscript for important intellectual content. All authors had full access to the data and approved the manuscript before it was submitted by the corresponding author.

## Supplementary Material

Supplemental data

## Figures and Tables

**Table 1:**
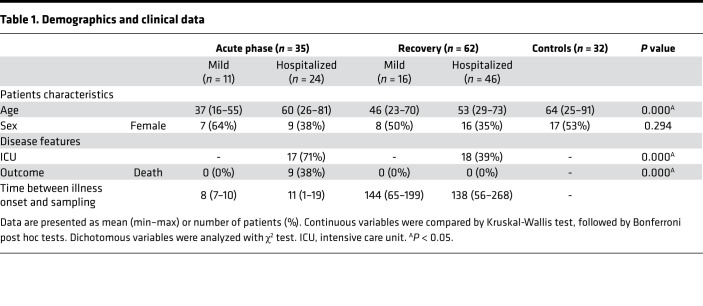
Demographics and clinical data

## Version 1 04/05/2022

In-Press Preview

## Version 2 05/09/2022

Electronic publication
