# Supplementary material for: Functional reprogramming of monocytes in patients with acute and convalescent severe COVID-19
Source: JCI Insight. 2022 Apr 5;7(9):e154183. doi: 10.1172/jci.insight.154183 (PMC9090263; doi:10.1172/jci.insight.154183)
Supplement: Supplemental data [file jciinsight-7-154183-s021.pdf]

## Supplemental figures

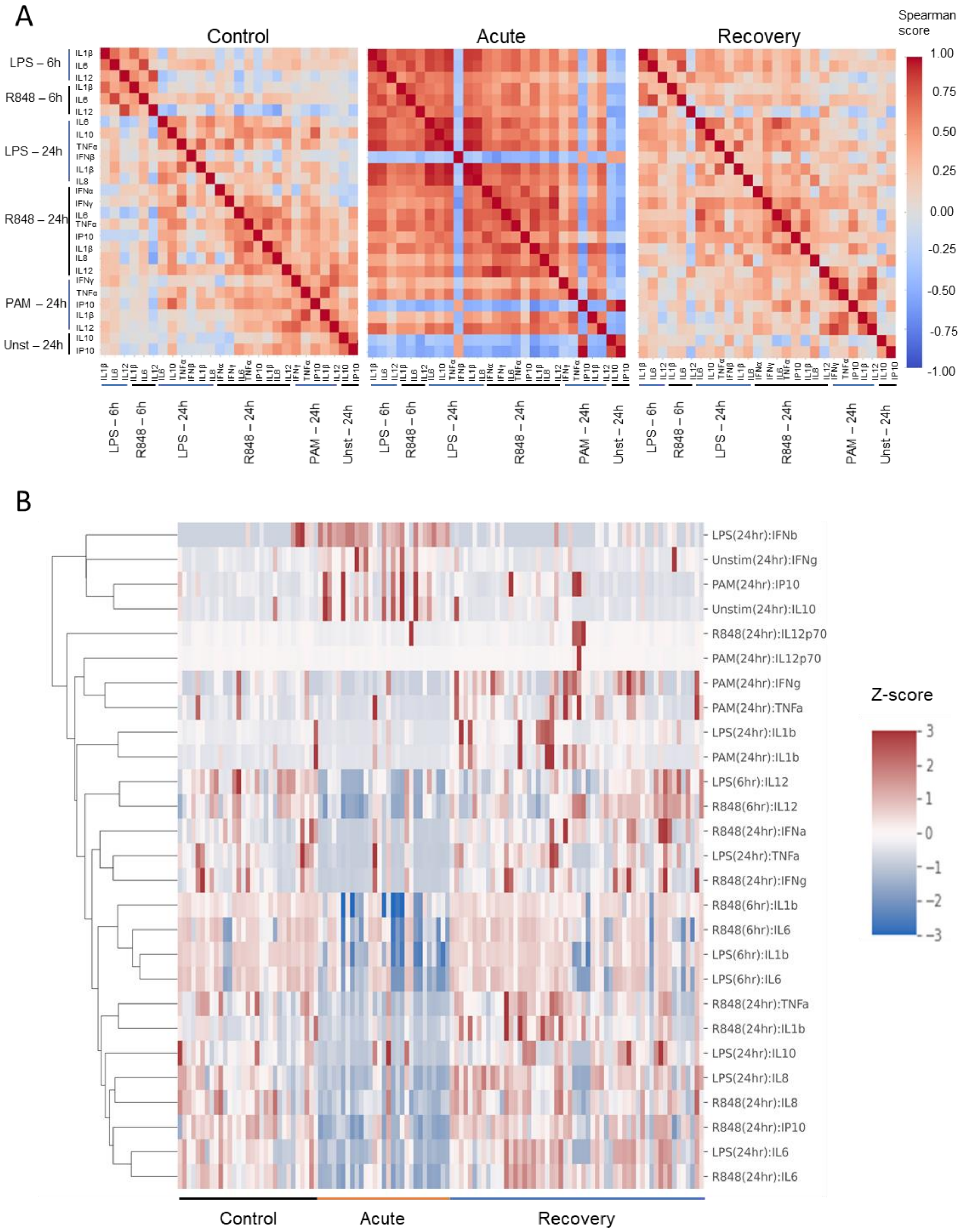


**Fig.S.1. The effect of COVID-19 on the capacity of monocytes to produce different cytokines is coordinated and global.** (A) Heatmap showing the correlations between the production of each cytokine under different stimulations, in controls or during acute and recovery phases. Spearman correlation coefficients were calculated between each pair of features. (B) Heatmap showing significantly different features between controls, acute and convalescent patients. The Z-score represents the deviation from the mean in each line.

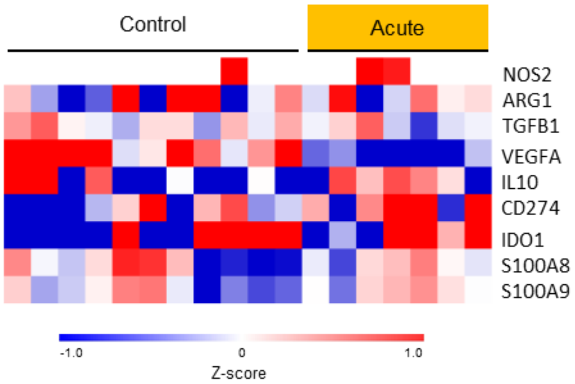


**Figure S2. No evidence of over-expression of genes associated with M-MDSC-like cells in CD14+ monocytes during acute severe COVID-19.** Heatmap of the expression of selected genes associated to M-MDSC-like cells in controls (n = 11) and during acute severe infection (n = 7).

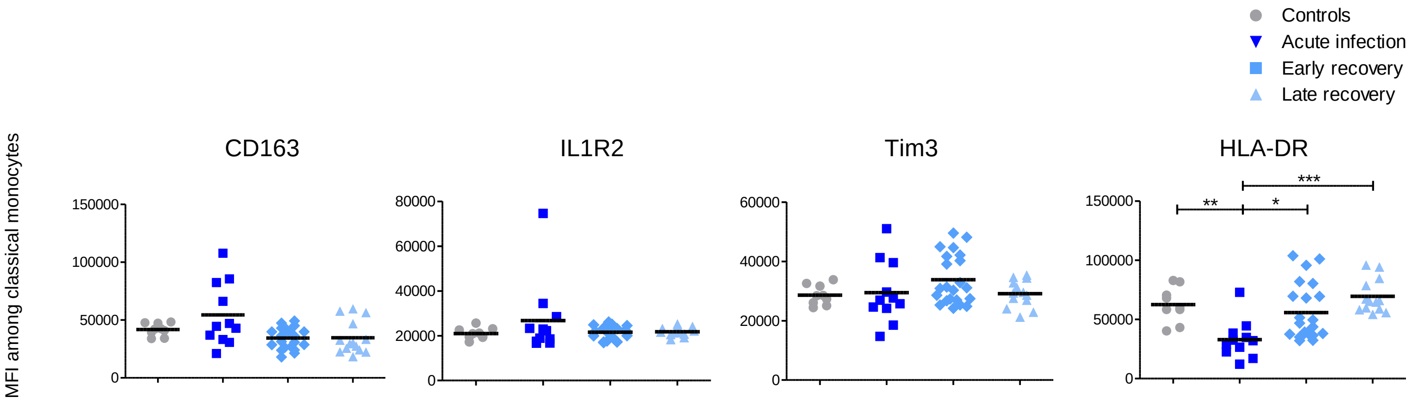


**Figure S3. Protein expression of selected surface markers identified as differentially expressed genes in RNA-Seq experiments in hospitalized patients.** Median fluorescence intensity of surface markers on $CD14^{+}$ monocytes in unstimulated whole blood from controls (n = 9), acute infection (n = 11), "early recovery" (n = 22) and "late recovery" phase (n = 13). Kruskal-Wallis test was performed to examine the statistical differences between groups, followed by Dunn's correction for multiple testing. *p <0.05, **p <0.01, ***p <0.001, ****p <0.0001
Each dot represents an individual donor and bar represents the mean value.

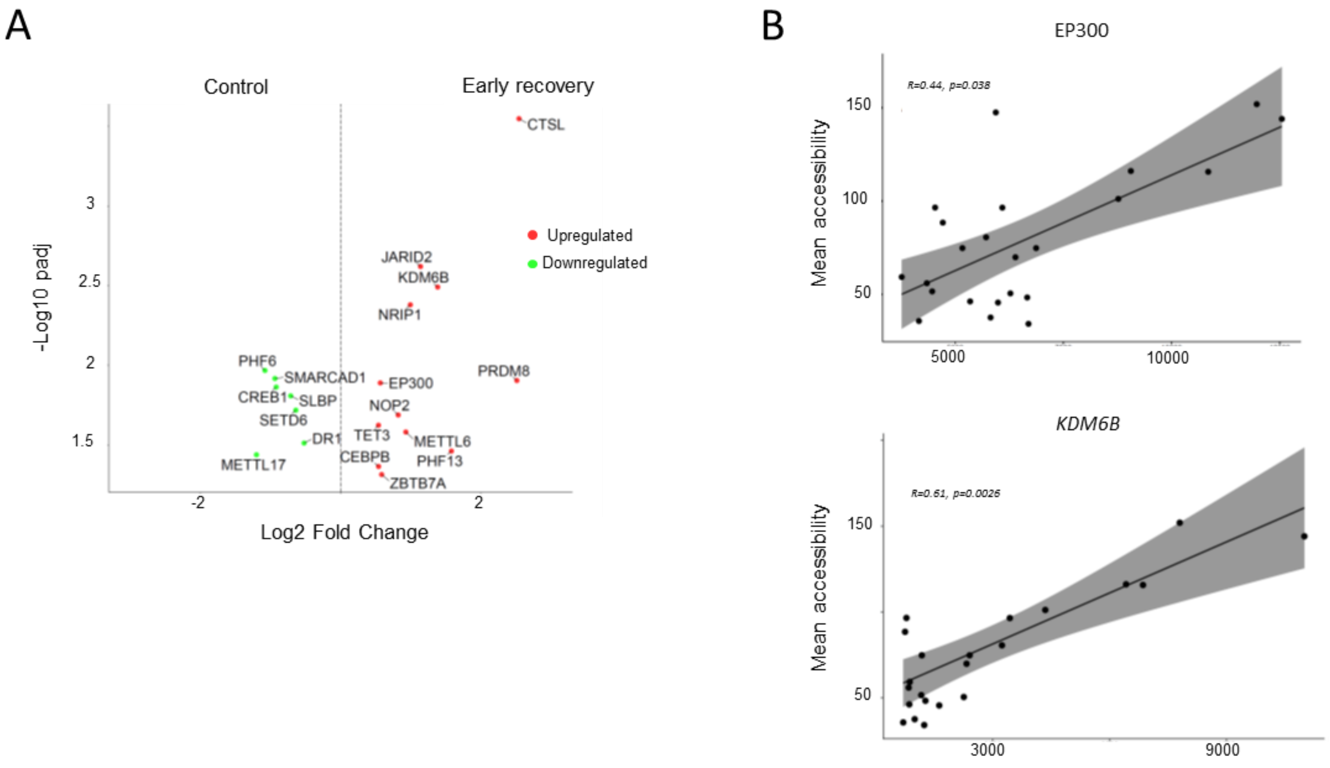


**Figure S4. Several epigenetic modulators are differentially expressed in monocytes from early convalescent patients.** (A) Volcano plot showing epigenetic modulators significantly up (red) or down (green) regulated in early recovered patients. (B)Scatter plot showing the correlation between AP-1/MAF module accessibility (expressed as a geometric mean of the top 70 most accessible regions) and RNA expression of histone acetyltransferase EP300 (top) and lysine-specific demethylase KDM6B (bottom). Correlation coefficient (R) and its p-value (p) are indicated.

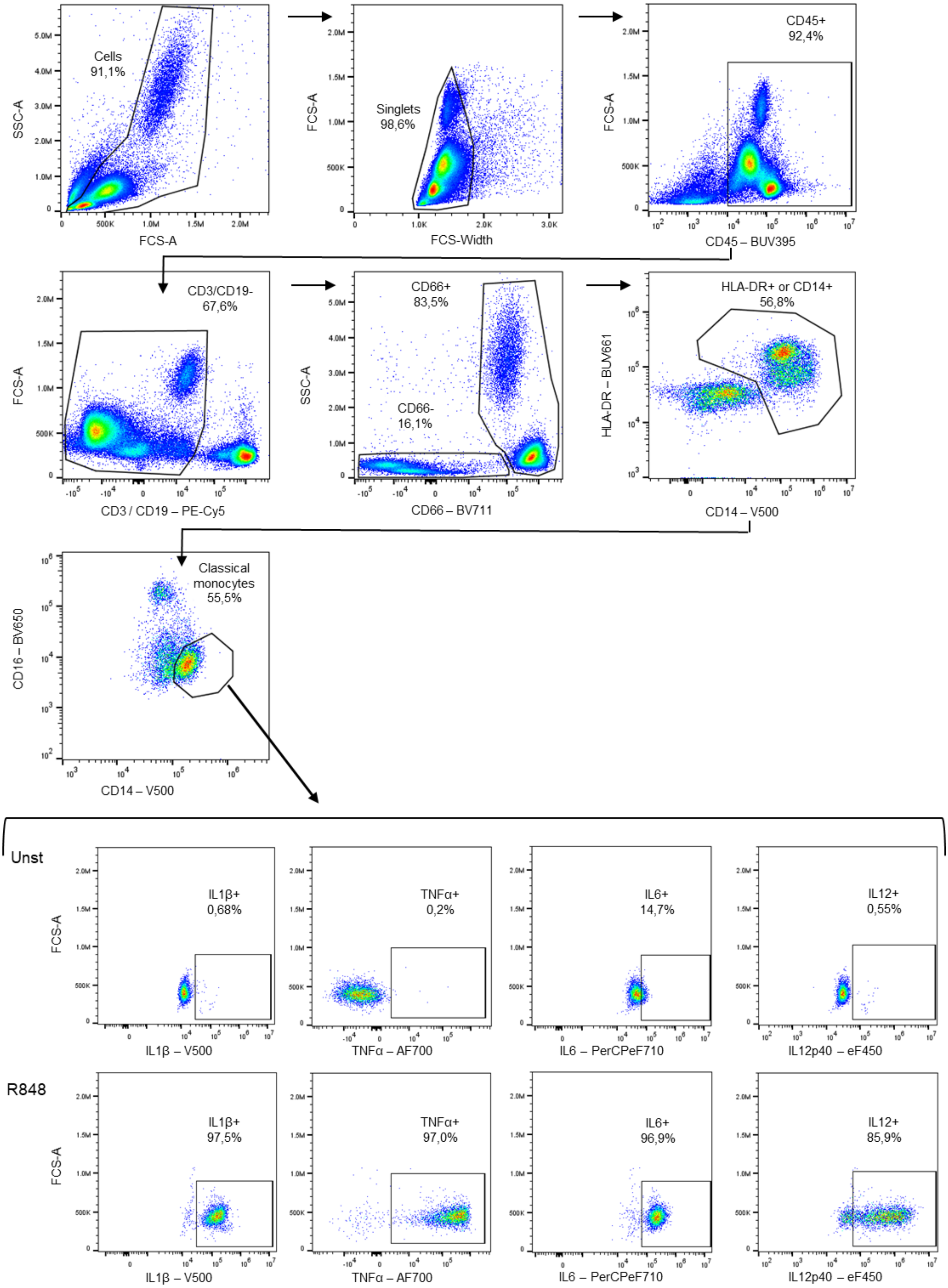


**Figure S5. Gating strategy for intracytoplasmic staining analyses by flow cytometry.** Plugin flow AI was used to increase the quality of the analysis. The following gating strategy is applied only on "good events" resulting from this process. Gating of intracytoplasmic cytokines is based on FMO controls for each of them.

Unst , unstimulated ; FMO, Fluorescence Minus One

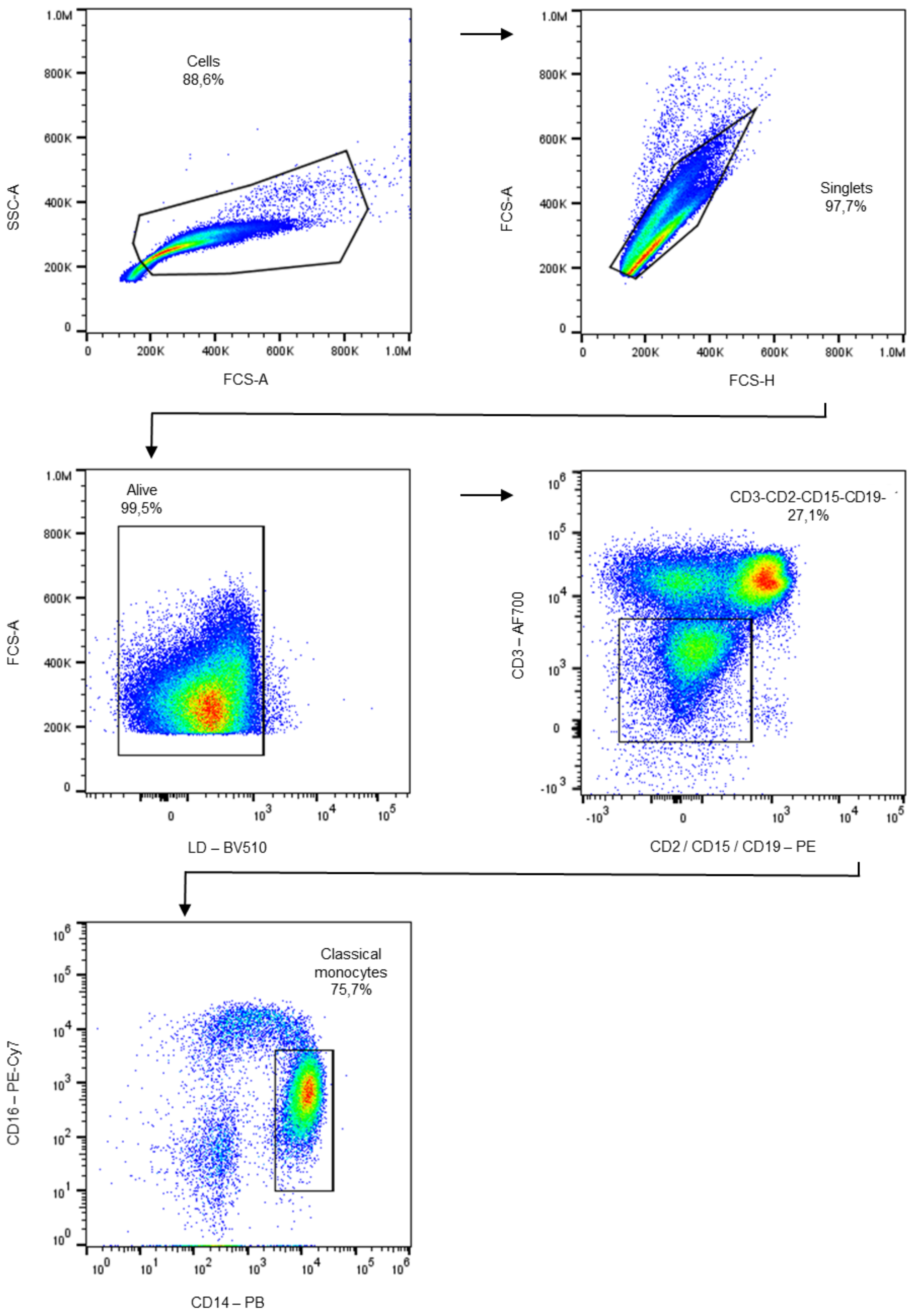


**Figure S6. Gating strategy for fluorescence-associated cell sorting of CD14+ monocytes from peripheral blood mononuclear cells.**

## Supplemental tables

| | | Acute phase (n = 35) | | | | Recovery (n = 62) | | | | *p* value |
|---|---|---|---|---|---|---|---|---|---|---|
| | | Mild (n = 11) | | Hospitalized (n = 24) | | Mild (n = 16) | | Hospitalized (n = 46) | | |
| *Patients characteristics* | | | | | | | | | | |
| Age [mean, (min-max)] | | 37 (16-55) | | 60 (26-81) | | 46 (23-70) | | 53 (29-73) | | 0,000* |
| Sex | Female | 7 | 64% | 9 | 38% | 8 | 50% | 16 | 35% | 0,294 |
| Ethnicity | Asian | - | - | 0 | 0% | 1 | 5% | 1 | 2% | 0,066 |
| | European | - | - | 11 | 48% | 12 | 75% | 23 | 50% | |
| | Latins | - | - | 0 | 0% | 0 | 0% | 1 | 2% | |
| | North-african | - | - | 12 | 52% | 1 | 5% | 13 | 30% | |
| | Sub-saharan-african | - | - | 0 | 0% | 2 | 11% | 8 | 19% | |
| Hypertension | | 2 | 18% | 12 | 50% | 4 | 27% | 19 | 41% | 0,231 |
| Diabetes | | 0 | 0% | 15 | 63% | 1 | 6% | 11 | 24% | 0,000* |
| Obesity | | 0 | 0% | 12 | 50% | 1 | 6% | 26 | 57% | 0,000* |
| | BMI (mean) | - | - | 33 (22-49) | | 25 (22-28) | | 33 (22-45) | | 0,025* |
| Dyslipidemia | | 0 | 0% | 7 | 29% | 3 | 19% | 9 | 20% | 0,252 |
| Heart disease | Ischemic disease | 2 | 18% | 1 | 4% | 1 | 6% | 2 | 4% | 0,367 |
| | Atrial fibrilation | 0 | 0% | 3 | 13% | 0 | 0% | 0 | 0% | 0,024* |
| Pulmonary disease | COPD | 0 | 0% | 2 | 8% | 1 | 6% | 1 | 2% | 0,537 |
| | Asthma | 0 | 0% | 2 | 8% | 2 | 13% | 8 | 17% | 0,394 |
| | Sleep apnea | 0 | 0% | 4 | 17% | 3 | 19% | 4 | 9% | 0,351 |
| Renal disease | Diabetic nephropathy | 0 | 0% | 0 | 0% | 1 | 6% | 5 | 11% | 0,256 |
| | Hypertensive nephrosclerosis | 0 | 0% | 1 | 4% | 0 | 0% | 0 | 0% | 0,380 |
| Malignancy | | 0 | 0% | 0 | 0% | 0 | 0% | 1 | 2% | 0,772 |
| Chronic immunosuppression | Immunosuppresive drugs | 0 | 0% | 2 | 8% | 0 | 0% | 3 | 7% | 0,544 |
| | HIV | 0 | 0% | 0 | 0% | 2 | 13% | 3 | 7% | 0,279 |
| Smoke | Active | 0 | 0% | 0 | 0% | 1 | 6% | 1 | 2% | 0,145 |
| | Past | 0 | 0% | 10 | 42% | 3 | 19% | 8 | 17% | |
| *Disease features* | | | | | | | | | | |
| Illness onset to admission | | - | - | 7,4 (0 - 14) | - | | | 7,8 (1-21) | | 0,931 |
| Positive nasopharyngeal RT-PCR | | 11 | 100% | 23 | 96% | 9 | 56% | 45 | 98% | 0,000* |
| Illness onset to RT-PCR | | - | - | 3,9 (-7 - 15) | | - | - | 7,3 (0-21) | | 0,007* |
| Radiological signs of COVID-19 | | - | - | 19 | 100% | 2 | 67% | 44 | 100% | 0,000* |
| Pulmonary embolism | Yes | - | - | 2 | 8% | 0 | 0% | 1 | 2% | 0,448 |
| | No | - | - | 6 | 25% | 2 | 13% | 10 | 22% | |
| | Not evaluated | - | - | 16 | 67% | 14 | 88% | 35 | 76% | |
| Lymphocytes (x10$^{3/mL}$) at admission | | - | - | 1,02 (0,29 - 2,02) | | - | | 1,14 (0,3 - 3,03) | | 0,010* |
| Neutrophils (x10$^3$/mL) at admission | | - | - | 7,24 (2,36 - 14,09) | | - | | 4,89 (2,22 - 9,98) | | 0,006* |
| CRP at admission | | - | - | 133 (26 - 530) | | - | | 105 (5 - 272) | | 0,001* |
| LDH at admission | | - | - | 502 (171 - 1249) | | - | | 392 (196 - 818) | | 0,000* |
| D-dimers at admision | | - | - | 3943 (233 - 35000) | | - | | 822 (286 - 2362) | | 0,957 |
| Ferritin at admission | | - | - | 1410 (68 - 4380) | | - | | 698 (59 - 2327) | | 0,063 |
| AKI stage | 0 | - | - | 15 | 63% | - | - | 34 | 74% | 0,023* |
| | 1 | - | - | 6 | 25% | - | - | 11 | 24% | |
| | 2 | - | - | 3 | 13% | - | - | 0 | 0% | |
| | 3 | - | - | 0 | 0% | - | - | 1 | 2% | |
| Specific treatments | Corticosteroid | 0 | 0% | 22 | 92% | 0 | 0% | 18 | 39% | 0,000* |
| | Antibiotic(s) | 0 | 0% | 15 | 63% | 1 | 6% | 21 | 46% | 0,000* |
| | Remdesivir | 0 | 0% | 2 | 8% | 0 | 0% | 2 | 4% | 0,524 |
| | Anti-interleukin therapies | 0 | 0% | 1 | 4% | 0 | 0% | 9 | 20% | 0,039* |
| | Antiviral therapy | 0 | 0% | 0 | 0% | 1 | 6% | 8 | 17% | 0,062 |
| | Hydroxychloroquine | 0 | 0% | 0 | 0% | 1 | 6% | 26 | 57% | 0,000* |
| | Convalescent plasma | 0 | 0% | 2 | 8% | 0 | 0% | 1 | 2% | 0,363 |
| Dialysis | | - | - | 1 | 4% | - | - | 2 | 4% | 0,754 |
| ICU | | - | - | 17 | 71% | | | 18 | 39% | 0,000* |
| Mechanical ventilation | | - | - | 12 | 50% | | | 12 | 26% | 0,001* |
| ECMO | | - | - | 5 | 21% | | | 2 | 4% | 0,025* |
| Length of MV | | - | - | 29 (6 - 75) | | | | 19 (3 - 66) | | 0,200 |
| Length of ICU stay | | - | - | 18,5 (1 - 82) | | | | 19 (4 - 73) | | 0,863 |
| Length of hospitalization | | - | - | 28 (4 - 152) | | | | 19 (3 - 111) | | 0,000* |
| Outcome | Death | 0 | 0% | 9 | 38% | 0 | 0% | 0 | 0% | 0,000* |
| Time between illness onset and sampling | | 8 (7-10) | | 11 (1 - 19) | | 144 (65 - 199) | | 138 (56 - 268) | | |
| Time between hospitalization and sampling | | - | - | 3 (0 - 10) | | - | - | 130 (47 - 262) | | |

**Table.S.1. Demographics and clinical data.** Data are presented as mean (min-max) or number of patients (%). Continuous variables were compared by Kruskal-Wallis test, followed by Bonferroni posttests. Dichotomous variables were analysed with Chi-square test.

AKI, acute kidney injury; ECMO, extracorporeal membrane oxygenation; ICU, intensive care unit; MV, mechanical ventilation

| | | Acute phase (n = 7) | | Recovery (n = 16) | | Controls (n=11) | | *p* value |
|---|---|---|---|---|---|---|---|---|
| *Patients characteristics* | | | | | | | | |
| Age [mean, (min-max)] | | 60 (46 - 75) | | 57 (41 - 73) | | 56 (45 - 65) | | 0,641 |
| Sex | Female | 3 | 43% | 5 | 31% | 5 | 50% | 0,622 |
| | Asian | 0 | 0% | 1 | 6% | - | - | 0,192 |
| | European | 4 | 57% | 8 | 50% | - | - | |
| Ethnicity | Latins | 3 | 43% | 2 | 13% | - | - | |
| | North-african | 0 | 0% | 5 | 31% | - | - | |
| | Sub-saharan-african | | | | | - | - | |
| Hypertension | | 3 | 43% | 10 | 63% | - | - | 0,565 |
| Diabetes | | 4 | 57% | 6 | 38% | - | - | 0,328 |
| Obesity | | 4 | 57% | 11 | 69% | - | - | 0,171 |
| | BMI (mean) | 29 (22 - 32) | | 32 (31 - 34) | | - | - | 0,136 |
| Dyslipidemia | | 2 | 29% | 4 | 25% | - | - | 0,697 |
| Heart disease | Ischemic disease | 1 | 14% | 2 | 13% | - | - | 0,856 |
| | Atrial fibrilation | 0 | 0% | 0 | 0% | - | - | . |
| Pulmonary disease | COPD | 0 | 0% | 0 | 0% | - | - | 0,697 |
| | Asthma | 0 | 0% | 1 | 6% | - | - | 0,746 |
| | Sleep apnea | 0 | 0% | 2 | 13% | - | - | 0,543 |
| Renal disease | Diabetic nephropathy | 0 | 0% | 3 | 19% | - | - | 0,219 |
| | Hypertensive nephrosclerosis | 0 | 0% | 0 | 0% | - | - | . |
| Malignancy | | 0 | 0% | 0 | 0% | - | - | . |
| Chronic immunosuppression | Immunosuppresive drugs | 1 | 14% | 1 | 6% | - | - | 0,735 |
| | HIV | 0 | 0% | 1 | 6% | - | - | 0,746 |
| Smoke | Active | 0 | 0% | 0 | 0% | - | - | 0,784 |
| | Past | 1 | 14% | 3 | 19% | - | - | |
| *Disease features* | | | | | | | | |
| Illness onset to admission | | 7 (4 - 13) | | 7 (3 - 14) | | - | - | 0,812 |
| Positive nasopharyngeal RT-PCR at hospital admission | | 7 | 100% | 16 | 100% | - | - | . |
| Illness onset to RT-PC | | 5 (0 - 11) | | 7 ( 0 - 14) | | - | - | 0,179 |
| Radiological signs of COVID-19 | | 5 | 100% | 16 | 100% | | | . |
| Pulmonary embolism | Yes | 0 | 0% | 0 | 0% | | | 0,104 |
| | No | 3 | 43% | 2 | 13% | - | - | 0,104 |
| | Not evaluated | 4 | 57% | 14 | 88% | - | - | |
| Lymphocytes (x10$^{3/mL)}$ at admission | | 1,04 (0,56 - 1,59) | | 1,01 (0,42 - 2,37) | | - | - | 0,815 |
| Neutrophils (x10$^{3}$/mL) at admission | | 6,19 (2,36 - 10,94) | | 5,06 (2,22 - 9,98) | | - | - | 0,570 |
| CRP at admission | | 132 (29 - 342) | | 109 (22 - 263) | | - | - | 0,738 |
| LDH at admission | | 672 (172 - 1249) | | 417 (256 - 724) | | - | - | 0,071 |
| D-dimers at admission | | 4527 (670 - 12442) | | 945 (323 - 2362) | | - | - | 0,151 |
| Ferritin at admission | | 2395 (68 - 4380) | | 943 (160 - 2327) | | - | - | 0,077 |
| AKI stage | 0 | 3 | 43% | 10 | 63% | - | - | 0,265 |
| | 1 | 3 | 43% | 6 | 38% | - | - | |
| | 2 | 1 | 14% | 0 | 0% | - | - | |
| | 3 | 0 | 0% | 0 | 0% | - | - | |
| Specific treatments | Corticosteroids | 7 | 100% | 1 | 6% | - | - | 0,000* |
| | Antibiotic(s) | 5 | 71% | 9 | 56% | - | - | 0,493 |
| | Remdesivir | 0 | 0% | 0 | 0% | - | - | . |
| | Anti-interleukin therapies | 0 | 0% | 5 | 31% | - | - | 0,095 |
| | Antiviral therapy | 0 | 0% | 3 | 19% | - | - | 0,219 |
| | Hydroxychloroquine | 0 | 0% | 13 | 81% | - | - | 0,000* |
| | Convalescent plasma | 0 | 0% | 0 | 0% | - | - | . |
| Dialysis | | 0 | 0% | 0 | 0% | - | - | . |
| ICU | | 7 | 100% | 9 | 56% | - | - | 0,036* |
| Mechanical ventilation | | 5 | 71% | 7 | 44% | - | - | 0,221 |
| ECMO | | 3 | 43% | 1 | 6% | - | - | 0,033* |
| Length of MV | | 16 (6 - 24) | | 17 (4 - 36) | | - | - | 0,909 |
| Length of ICU stay | | 12 (1 - 24) | | 21 (5 - 46) | | - | - | 0,243 |
| Length of hospitalization | | 23 (16 - 42) | | 26 (5 - 73) | | - | - | 0,969 |
| Outcome | Death | 4 | 57% | 0 | 0% | - | - | 0,001* |
| Time between illness onset and sampling | | 11 (4 - 19) | | 159 (68 - 246) | | - | - | 0,000* |
| Time between hospitalization and sampling | | 4 (0 - 10) | | 152 (61 - 237) | | - | - | 0,000* |

**Table.S.2. Demographics and clinical data of the subset of patients used for molecular analyses.** Data are presented as mean (min-max) or number of patients (%). Continuous variables were compared by Kruskal-Wallis test, followed by Bonferroni posttests. Dichotomous variables were analysed with Chi-square test.

AKI, acute kidney injury; ECMO, extracorporeal membrane oxygenation; ICU, intensive care unit; MV, mechanical ventilation

| *Antibody* | *Clone* | *Fluorochrome* | *Source* | *Catalog number* |
|---|---|---|---|---|
| CD45 | HI30 | BUV395 | BD Biosciences | 563792 |
| CD3 | UCHT1 | PECy5 | BD Biosciences | 555334 |
| CD19 | HIB19 | PECy5 | BD Biosciences | 15-0199-42 |
| CD66 | B1.1 | BV711 | BD Biosciences | 740805 |
| HLA-DR | G46-6 | BUV661 | BD Biosciences | 612980 |
| CD14 | M5E2 | V500 | BD Biosciences | 561391 |
| CD11c | SHCL-3 | APC | BD Biosciences | 333144 |
| CD226 | DX11 | BUV496 | BD Biosciences | 749935 |
| CD83 | HB15e | BV786 | BD Biosciences | 565336 |
| Tim3 (CD366) | 7D3 | BB700 | BD Biosciences | 747957 |
| CD163 | GHI/6.1 | PE | BD Biosciences | 560933 |
| CD64 | 10.1 | PE-CF594 | BD Biosciences | 565389 |
| CD86 | 2331 | AF700 | BD Biosciences | 561124 |
| CD69 | FN50 | APC-Cy7 | BD Biosciences | 557756 |
| CD16 | 3G8 | BV650 | Biolegend | 302042 |
| PDL1 (CD274) | 29E.2A3 | BV421 | Biolegend | 329714 |
| CD40 | SC3 | FITC | Biolegend | 334306 |
| CD123 | 6H6 | PECy7 | ThermoFisher | 25-1239-42 |
| IL1R2 | 34141 | FITC | ThermoFisher | MA523662 |
| TNFα | Mab11 | AF700 | ThermoFisher | 56-7349-42 |
| IL1β | CRM56 | FITC | ThermoFisher | 11-7018-42 |
| IL12/23p40 | C8.6 | eFluor450 | ThermoFisher | 48-7129-42 |
| IL6 | MQ2-13A5 | PerCpeFluor710 | ThermoFisher | 46-7069-42 |

**Table S3. List of antibodies used for flow cytometry staining.**
